# Trends in Nationally Notifiable Infectious Diseases in Humans and Animals during COVID-19 Pandemic, South Korea

**DOI:** 10.3201/eid3006.231422

**Published:** 2024-06

**Authors:** Taehee Chang, Sung-il Cho, Dae sung Yoo, Kyung-Duk Min

**Affiliations:** Seoul National University, Seoul, South Korea (T. Chang, S. Cho);; Institute of Health and Environment, Seoul (S. Cho);; Chonnam National University, Gwangju, South Korea (D. Yoo);; Chungbuk National University, Cheongju, South Korea (K.-D. Min)

**Keywords:** COVID-19, Nationally notifiable, infectious diseases, coronavirus disease, SARS-CoV-2, severe acute respiratory syndrome coronavirus 2, viruses, respiratory infections, zoonoses, vaccine-preventable diseases, South Korea

## Abstract

We investigated trends in notifiable infectious diseases in both humans and animals during the COVID-19 pandemic in South Korea and compared those data against expected trends had nonpharmaceutical interventions (NPIs) not been implemented. We found that human respiratory infectious diseases other than COVID-19 decreased by an average of 54.7% after NPIs were introduced. On the basis of that trend, we estimated that annual medical expenses associated with respiratory infections other than COVID-19 also decreased by 3.8% in 2020 and 18.9% in 2021. However, human gastrointestinal infectious diseases and livestock diseases exhibited similar or even higher incidence rates after NPIs were instituted. Our investigation revealed that the preventive effect of NPIs varied among diseases and that NPIs might have had limited effectiveness in reducing the spread of certain types of infectious diseases. These findings suggest the need for future, novel public health interventions to compensate for such limitations.

The global COVID-19 pandemic, caused by SARS-CoV-2, dramatically disrupted the lives of persons around the world, resulting in record numbers of cases and deaths (*1*). In the early stages of the pandemic, public health measures primarily consisted of nonpharmaceutical interventions (NPIs), such as social distancing, mask wearing, and contact tracing. NPIs are effective in mitigating the epidemic curves in various contexts, even without vaccines or specific treatments targeting the pathogen (*2*–*4*). Since March 2020, stringent public health measures have been implemented nationwide in South Korea, effectively suppressing the spread of COVID-19 (*5*–*7*).

The effects of NPIs are not necessarily limited to COVID-19. Because NPIs reduce effective contacts within a population, such measures can also mitigate other respiratory infectious diseases (*5*,*8*,*9*). Likewise, implementation of social distancing measures (e.g., restrictions on social gatherings in restaurants) and improved personal hygiene practices can reduce occurrence rates of gastrointestinal diseases (*9*,*10*). Mitigation measures targeting COVID-19 might even extend beyond human diseases, potentially reducing risks for infectious diseases in animals (*11*–*13*). Human movement restrictions and the global economic crisis have greatly disrupted farming operations, veterinary services, wildlife surveillance, and zoonotic disease control, broadly influencing animal health and welfare (*11*,*12*). Those effects could contribute to outbreaks of major zoonotic diseases, such as brucellosis and bovine tuberculosis in animal populations, increasing the risk for zoonotic spillover (*13*).

Research on the effects of NPIs implemented during the COVID-19 pandemic on other infectious diseases in South Korea has found that reductions in respiratory infections coincided with social distancing interventions (*14*–*20*). However, the effects of NPIs on gastrointestinal diseases were inconsistent. Studies revealed a notable reduction in viral gastrointestinal infections but no marked decrease in bacterial infections, such as those caused by *Campylobacter* spp., *Clostridium perfringens*, and *Salmonella* spp. (*10*,*18*,*20*). Decreases in viral gastrointestinal diseases were attributed to the primary transmission route being fecal–oral contamination or direct contact between persons. In contrast, bacterial gastrointestinal infections are mainly foodborne illnesses attributable to consuming contaminated food or water (*21*). 

We focused on nationally notifiable infectious diseases in humans and livestock, using data collected after 2020. We sought to quantify the effect of nationally implemented NPIs in South Korea on the trends of infectious diseases other than COVID-19, to evaluate the benefits and drawbacks of NPIs, and to provide scientific evidence informing future health policy decisions aimed at mitigating various types of infectious diseases. We focused our study on the period from 2016 through the end of 2021, a period of social distancing in South Korea instituted in response to the COVID-19 pandemic. In the first half of 2022, NPIs were tapered back, as were their potential attenuation effects. To quantify the effect of NPIs in South Korea, we built time series models (*22*) for 6 respiratory human infectious diseases, 4 human gastrointestinal diseases, and 2 livestock diseases. 

## Methods

### Study Design

We retrospectively analyzed the effect of COVID-19–associated NPIs on incidence of infectious diseases in South Korea. We used the following criteria in selecting target infectious diseases from among the nationally notifiable diseases: human infectious diseases with a principal mode of transmission that is respiratory (airborne or droplets) or gastrointestinal (foodborne or via fecal–oral route); animal infectious diseases with a risk for zoonotic transmission; and diseases with an annual average incidence >100 cases. Acknowledging that the effects of NPIs might not be fully applicable to certain infectious diseases that require isolation after diagnosis or symptom onset, we nonetheless theorized that implementation of NPIs in a population can potentially suppress the spread caused by asymptomatic carriers or infectious persons before isolation. We therefore included such diseases as target infectious diseases in this study. We defined the preintervention period as January 2016–February 2020 and the intervention period as March 2020–December 2021. From May 2022 onward, the outdoor mask mandate was conditionally lifted.

We used autoregressive integrated moving average (ARIMA) models to forecast disease incidence during the intervention period on the basis of patterns in the preintervention period and compared predicted values with observed values in the intervention period. The time-dependent reproduction number (R_t_) affords an optimal understanding of the transmission dynamics of respiratory infectious diseases (*23*). Therefore, we calculated R_t_ values for respiratory infectious diseases during time series forecasting.

Previous studies investigating the effects of COVID-19 and NPIs on other diseases suggest that the reduced burden of target diseases during the early stages of the COVID-19 pandemic could be attributed to pandemic-related decreases in healthcare utilization and disease diagnoses (*19*). To adjust for the effect of decreased healthcare utilization, we collected information on annual hospital visits (*24*) and annual health insurance claims (*25*) (Appendix 1 Table 1), and used those numbers as denominators when calculating disease incidence. When calculating incidence rates per population, we collected annual midyear population data for each year in South Korea (*26*). We also obtained total annual medical expenses associated with each infectious disease to evaluate how changes in disease occurrence after NPI implementation might have affected the overall disease burden (*27*). We calculated annual medical expenses per case using Health Insurance Review and Assessment Service data from 2018–2021 (*27*). Then, we multiplied expenses per case by the estimated and observed cases of each disease to determine the model-based medical costs and observation-based values for each disease. We compared those values when assessing changes in the overall disease burden.

### Social Distancing Measures

**Figure 1:**
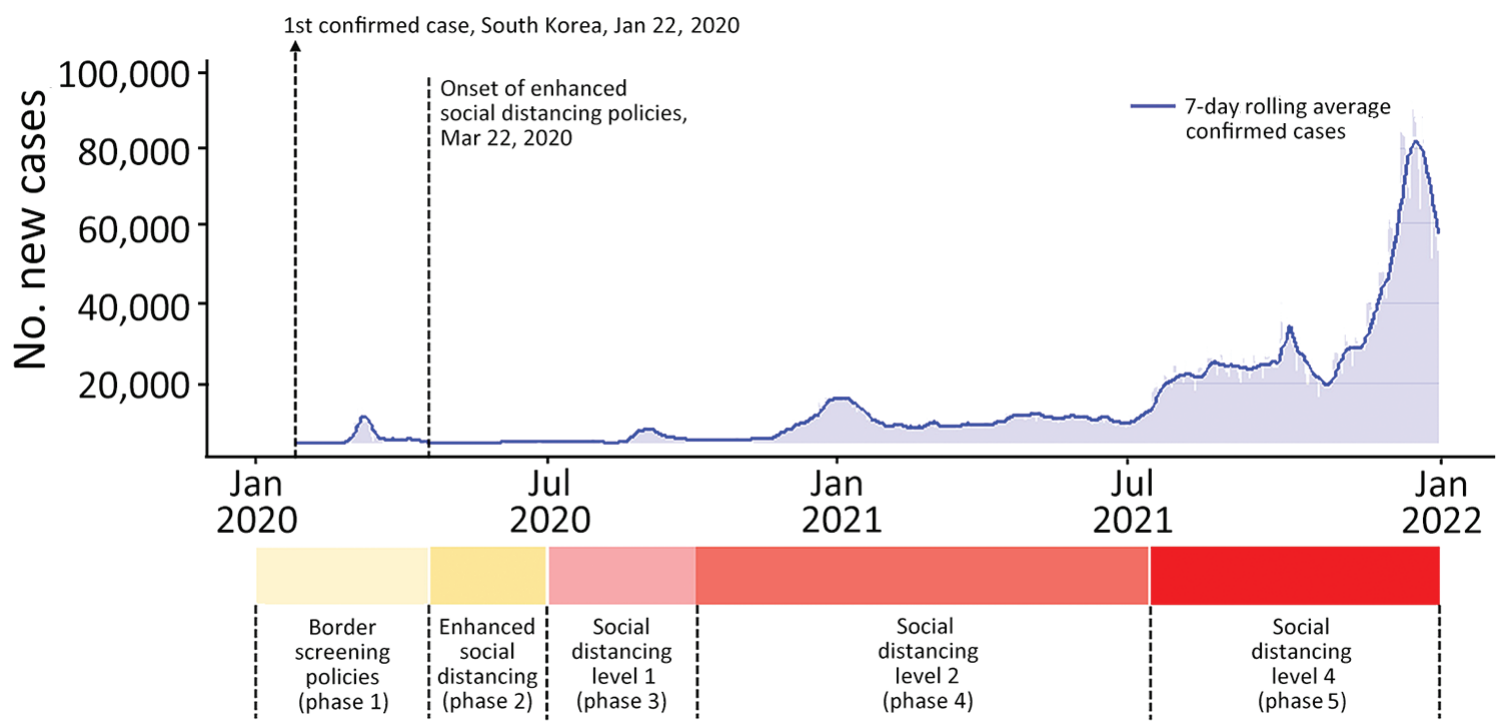
Daily numbers of confirmed cases and 7-day rolling average numbers of COVID-19 cases in a study of trends in nationally notifiable infectious diseases in humans and animals during the COVID-19 pandemic, South Korea. Phase and level information is provided in Tables 2 and 3. The levels of nonpharmaceutical interventions depicted in this figure are those of the 4-tier system implemented in July 2021.

In February 2020, in response to the COVID-19 outbreak in China, South Korea implemented a universal mask mandate and recommended physical distancing (Table 1). After the increase in COVID-19 cases in South Korea, nationwide social distancing requirements were implemented with various restrictions starting in March 2020 (*28*). During the initial phase of the COVID-19 pandemic, the Distancing in Daily Life strategy was put into practice in South Korea (*29*). After multiple outbreaks occurred near metropolitan areas, the Distancing in Daily Life strategy was restructured on June 28, 2020, into a 3-tier social distancing system that consisted of levels 1, 2, and 3 (Appendix 1 Table 2) (*1*). In November 2020, the social distancing system was reorganized into a 5-tier structure that consisted of levels 1, 1.5, 2, 2.5, and 3 (Appendix 1 Table 3). Subsequently, in July 2021, the system was modified to a 4-tier structure that consisted of levels 1, 2, 3, and 4 (Appendix 1 Table 4) (*29*). In this study, we documented the policy changes based on the 4-tier structure; we did not consider any rapid changes within short periods (e.g., 1–2 weeks or 1 month) because they might not have been adequately effective (Figure 1).

**Table 1:** Changes in social distancing policies used in a study of trends in nationally notifiable infectious diseases in humans and animals during COVID-19 pandemic, South Korea

Time period and social distancing level	General description of terms
June 2020–November 2020	
1	Distancing in daily life
2	Moderate social distancing
3	Intensive social distancing
November 2020–July 2021	
1	Distancing in daily life
1.5	Local outbreak initiation
2	Rapid local spread, nationwide spread initiation
2.5	Nationwide outbreak intensification
3	Nationwide major epidemic
July 27, 2021 onward	
1	Sustained suppression phase
2	Regional outbreak
3	Regional epidemic
4	Nationwide epidemic

### Data Acquisition

We collected data on the weekly and monthly domestic cases of nationally notifiable infectious diseases from the Infectious Disease Portal of the Korea Disease Control and Prevention Agency (*30*). To minimize sampling bias and ensure that our analysis was robust despite the COVID-19–related decrease in healthcare utilization, we focused on only infectious diseases listed in the mandatory surveillance system. We collected records on cases of 6 respiratory infectious diseases (varicella, pertussis, mumps, invasive pneumococcal disease [IPD], scarlet fever, and tuberculosis [TB]); 4 gastrointestinal diseases (typhoid fever, shigellosis, hepatitis A, and enterohemorrhagic Escherichia coli [EHEC]) that occurred during January 2016–December 2021 (Appendix 1 Table 5).

We collected data from the Korea Animal Health Integrated System in investigating animal diseases with zoonotic potential (*31*). The Korea Animal Health Integrated System is a comprehensive system operated by the Animal and Plant Quarantine Agency that integrates and provides nationwide information on livestock diseases. We selected 2 livestock diseases (cattle TB and cattle brucellosis) and collected occurrence data for January 2016–December 2021 (Appendix 1 Table 5). We focused on cattle TB and cattle brucellosis because those diseases pose risks for human infection and annual cases are numerous. We then investigated the effects of NPIs. We also collected data relating to the annual number of livestock and the annual scale of livestock farming (*32*) when calculating incidence rates relative to the livestock population.

### R_t_ Estimation

R_t_ represents the average number of new infections generated by an infected person during the infectious period. This time- and context-specific measure is frequently used to assess the transmissibility of a pathogen during an outbreak. Therefore, in this study, we used R_t_ to reflect the dynamics of respiratory infectious diseases accurately when estimating the effectiveness of NPIs. We estimated the incidence levels of infectious diseases within the population and assessed trends in disease occurrence, except for TB, on the basis of R_t_. Although TB is a respiratory infectious disease, we did not calculate R_t_ because of the complex transmission routes and long latent period; rather, we used reported cases for time series forecasting of tuberculosis. The calculation of R_t_ was based on examples from previous studies (*23*,*33*–*35*) (Appendix 2).

### Time Series Analysis

The ARIMA model is a time series forecasting technique that incorporates elements of an autoregressive moving average when making predictions (*22*). Autoregression of time series data shows how past values influence the current value. The moving average indicates how prediction errors affect the current value. This component adjusts for irregularities in the time series by using past prediction errors to correct the current value. The model is commonly used to predict the short-term impacts and trends of acute infectious diseases (*9*,*22*). Time series forecasting based on the Box-Jenkins method features 4 steps: identification, estimation, diagnostic checking, and forecasting (*36*). We used those steps when making predictions. In addition, we conducted out-of-sample validation to confirm the predictive performance of the model and ensure that the model had not overfitted the training data. In the validation process, we used data from 2015–2018 as training data and predicted and compared the trends for 2019 with the observed values (Appendix 2). We performed all data processing and analyses using R version 4.2.2 (The R Foundation for Statistical Computing, https://www.r-project.org). 

## Results

### Incidences of Human Respiratory Diseases

After nationwide social distancing measures were put in place in South Korea in March 2020 (Figure 1), considerable decreases in the weekly reported case numbers for human respiratory diseases were observed (Table 2). The mean weekly incidence levels (cases/1 million population) for 2016–2019 varied for each disease: varicella, 30.11; pertussis, 0.18; mumps, 6.95; IPD, 0.21; scarlet fever, 5.57; and TB, 13.13. However, after implementation of NPIs, the mean weekly incidence levels for 2020–2021 substantially decreased, and showed slight variations among the phases: varicella, 12.09; pertussis, 0.05; mumps, 4.00; IPD, 0.13; scarlet fever, 0.80; and TB, 9.16. The annual medical expenses associated with respiratory infectious diseases decreased by 3.77% in 2020, compared with the value calculated using the average estimated incidence; the value decreased by an additional 18.91% in 2021 (Table 3). Whereas medical expenses related to respiratory infectious diseases exhibited an overall decreasing trend, TB-related expenses showed a slight increase in 2020; scarlet fever–related expenses also exhibited a slight increase in 2021.

**Table 2:** Weekly average incidences of diseases included in study of trends in nationally notifiable infectious diseases in humans and animals during COVID-19 pandemic, South Korea*

Disease	2016–2019	2020–2021					
		Overall	Phase 1	Phase 2	Phase 3	Phase 4	Phase 5
Human, cases/1 million population							
Respiratory diseases							
Varicella	30.11	12.09	25.76	9.29	9.31	8.27	7.81
Pertussis	0.18	0.05	0.17	0.03	0.01	0.01	0.01
Mumps	6.95	4.00	3.89	4.28	4.34	3.29	4.21
Invasive pneumococcal disease	0.21	0.13	0.28	0.11	0.08	0.1	0.1
Scarlet fever	5.57	0.80	2.12	0.76	0.59	0.34	0.21
Tuberculosis	13.13	9.16	10.02	9.17	9.31	8.94	8.34
Gastrointestinal or enteroviral diseases							
Typhoid	0.04	0.03	0.03	0.05	0.04	0.02	0.03
Shigellosis	0.02	0.02	0.03	0.04	0.01	0.01	0.01
Hepatitis A	3.02	1.91	1.37	1.58	1.59	2.48	2.53
Enterohemorrhagic *Escherichia coli*	0.05	0.12	0.04	0.23	0.21	0.06	0.06
Veterinary, cases/100,000 animals							
Bovine tuberculosis	2.19	1.29	1.04	1.82	1.43	1.27	0.91
Bovine brucellosis	0.49	0.61	0.41	0.51	0.55	0.83	0.74

*Detailed information on social distancing policies is provided in Appendix 1 Tables 2–4. Phase 1, border screening policies, February 20–March 21, 2020; phase 2, enhanced social distancing, March 22–June 27, 2020; phase 3, social distancing level 1, June 28–August 22, 2020; phase 4, social distancing level 2, August 23, 2020–July 26, 2021; phase 5, social distancing level 4, July 27–December 31, 2021.

**Table 3:** Annual medical expenses due to infectious diseases in a study of trends in nationally notifiable infectious diseases in humans and animals during COVID-19 pandemic, South Korea*

Diseases	Expenses, in million USD									
	Observed, 2018	Observed, 2019	Estimated, 2020			Observed, 2020 (% difference)†	Estimated, 2021			Observed, 2021 (% difference)‡
			Lower 95%	Average	Upper 95%		Lower 95%	Average	Upper 95%	
Respiratory										
Varicella	5,399	5,590	3,704	7,153	13,548	2,675 (−62.61)	2,692	6,559	16,001	1,971 (−69.95)
Pertussis	544	298	44	380	1,171	42 (−88.98)	13	409	1,900	6 (−98.65)
Mumps	1,283	1,445	1,203	1,552	2,189	790 (−49.08)	1,066	1,532	2,203	775 (−49.42)
IPD	1,988	1,686	1,305	2,920	6,437	1,360 (−53.41)	1,305	3,370	8,717	1,116 (−66.87)
Scarlet fever	1,283	691	182	465	947	317 (−31.80)	28	124	567	154 (24.27)
Tuberculosis	61,241	63,535	42,695	48,705	55,593	53,681 (10.21)	48,536	59,956	74,117	54,324 (−9.39)
Subtotal	71,738	73,244	49,134	61,174	79,886	58,864 (−3.77)	53,640	71,951	103,505	58,345 (−18.91)
Gastrointestinal										
Typhoid	153	41	6	17	62	27 (89.81)	5	22	102	41 (90.625)
Shigellosis	36	49	6	22	40	12 (−46.86)	3	21	126	15 (−28.06)
Hepatitis A	2,248	27,297	1,477	6,193	22,857	4,820 (−22.17)	1,466	9,407	60,328	9,769 (3.85)
EHEC	120	157	94	176	275	364 (106.61)	86	184	388	244 (33.06)
Subtotal	2,557	27,544	1,583	6,408	23,234	5,223 (−18.49)	1,560	9,633	60,945	10,070 (4.53)
Total	74,295	100,788	50,717	67,582	103,120	64,087 (−5.17)	55,201	81,584	164,450	68,415 (−16.14)

*EHEC, enterohemorrhagic *Escherichia coli*; IPD, invasive pneumococcal disease; USD, US dollars. †Difference from average estimates for 2020. ‡Difference from average estimates for 2021.

**Figure 2:**
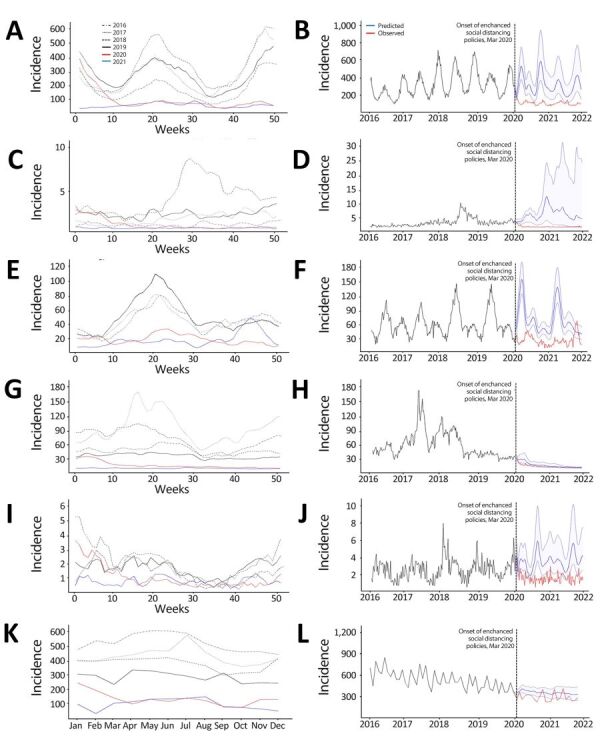
Incidence trends (cases/1 million population) in 6 nationally notifiable respiratory infectious diseases in humans before and during the COVID-19 pandemic, South Korea. A, C, E, G, I) Weekly incidence levels for various diseases retrieved from the national surveillance system for 2016–2019 versus 2020–2021. B, D, F, H, J) Observed and predicted weekly incidence levels during 2016–2021. A, B) varicella; C, D) pertussis; E, F) mumps; G, H) scarlet fever; I, J) invasive pneumococcal diseases. K) Monthly incidence levels of tuberculosis; L) observed and predicted monthly incidence levels of tuberculosis.

**Figure 3:**
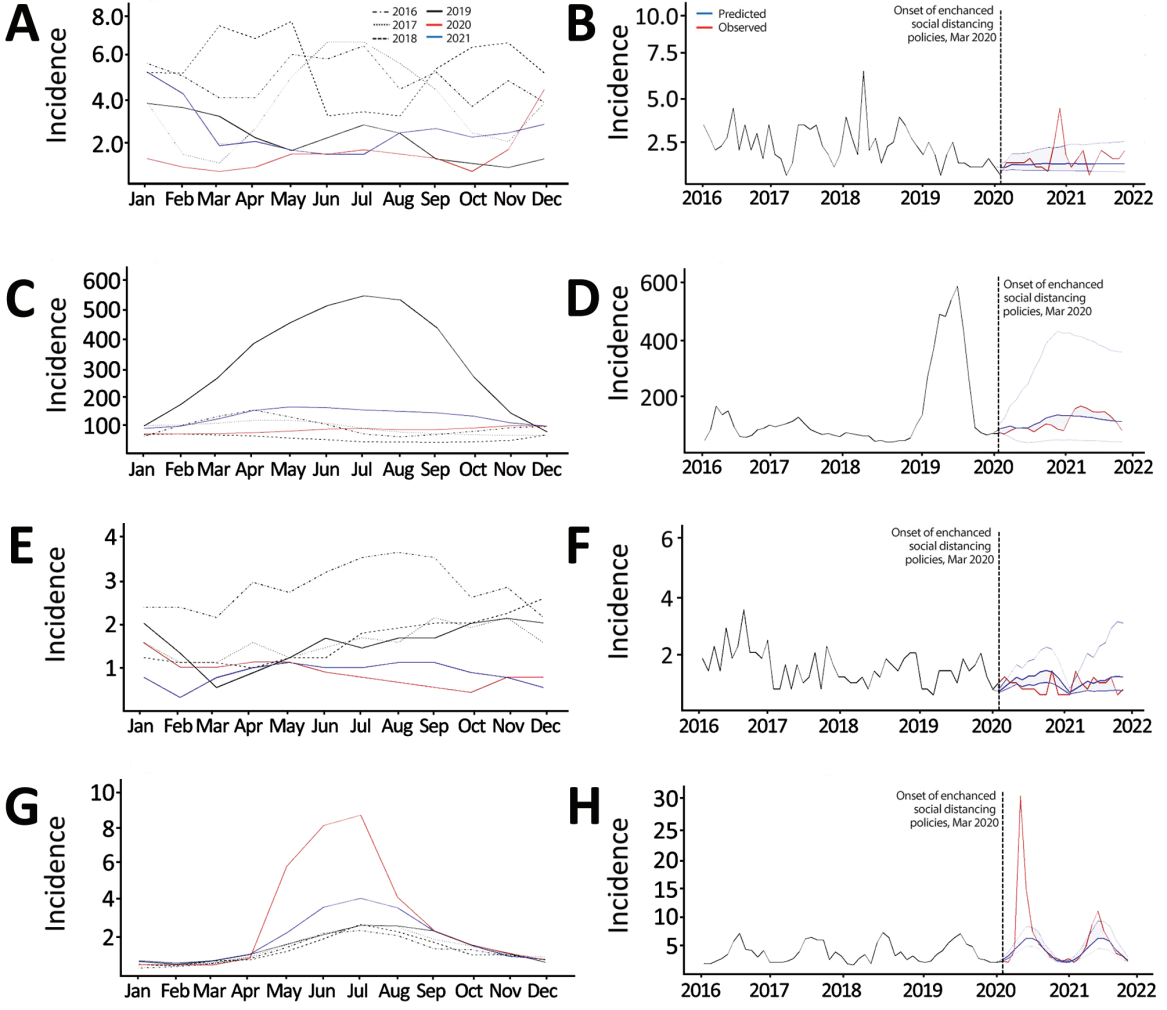
Incidence trends (cases/1 million population) in 4 nationally notifiable gastrointestinal infectious diseases in humans before and during the COVID-19 pandemic, South Korea. A, C, E, G) Monthly incidence levels retrieved from the national surveillance system for 2016–2019 versus 2020–2021; B, D, F, H) observed and predicted monthly incidence levels during 2016–2021. A, B) typhoid; C, D) hepatitis A; E, F) shigellosis; and G, H) *Escherichia coli*.

**Figure 4:**
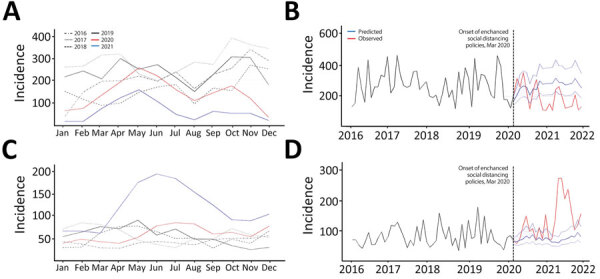
Incidence trends (cases/100,000 animals) in 2 nationally notifiable zoonotic infectious diseases in animals before and during the COVID-19 pandemic, South Korea. A, C) Monthly incidence levels retrieved from the national surveillance system for 2016–2019 versus 2020–2021; B, D) observed and predicted monthly incidence levels during 2016–2021. A, B) Bovine tuberculosis; C, D) bovine brucellosis.

ARIMA models (Appendix 1 Tables 7–19, Figures 1–12) showed that, except for TB, the actual incidence of diseases examined during the intervention period were substantially lower than the predicted incidence (Figure 2, panels A–J). The incidence levels of TB were lower than the predicted values, but the average predicted values were within 25.6% of the numbers of reported cases. Although the average predicted values decreased, compared with predicted values, after the implementation of social distancing measures, the observed incidence remained at levels similar to the predicted values from the second half of 2020 (Figure 2, panels K, L).

### Incidence of Human Gastrointestinal Diseases

Unlike for respiratory infectious diseases, the incidence of the 4 gastrointestinal diseases did not exhibit remarkable decreases after the implementation of NPIs (Table 2). The mean weekly incidence levels (cases/1 million population) for 2016–2019 varied among the diseases: typhoid, 0.04; shigellosis, 0.02; hepatitis A, 3.02; and EHEC, 0.05. Although we observed slight variations among the phases, the mean weekly incidence levels for 2020–2021 after implementation of social distancing measures were as follows: typhoid, 0.03; shigellosis, 0.02; hepatitis A, 1.91; and EHEC, 0.12. Annual medical expenses associated with gastrointestinal infectious diseases decreased by 18.49% in 2020, compared with the value calculated by using the average estimated incidence; the value increased by 4.53% in 2021 (Table 3). The trend in medical expenses associated with gastrointestinal infectious diseases varied depending on the specific condition; different trends were observed for each disease.

ARIMA models (Appendix 1 Tables 7, 20–27, Figures 13–20) Showed that the observed incidence levels of gastrointestinal diseases were generally close to the average predicted values (Figure 3). However, unexpected outbreaks of typhoid and EHEC occurred, resulting in higher observed incidence levels than predicted (Figure 3, panels A, B, G, H).

### Incidence of Zoonotic Diseases in Animals

Comparisons of the periods before and after implementation of NPIs revealed contrasting patterns for bovine TB and bovine brucellosis (Table 2). The mean weekly incidence (cases/100,000 cattle) for 2016–2019 varied between the diseases: bovine TB, 2.19; and bovine brucellosis, 0.49. Although slight variations were observed among the phases, the mean weekly incidence levels for 2020–2021 after implementation of social distancing measures were as follows: bovine TB, 1.29; bovine brucellosis, 0.61.

ARIMA models (Appendix 1 Tables 7, 28–31, Figures 21–24) showed that incidence levels of bovine TB were noticeably lower than expected from the end of 2020 (Figure 4, panels A, B). In contrast, the incidence of bovine brucellosis rapidly increased and reached a record high in June 2021 (Figure 4, panels C, D).

## Discussion

We used national surveillance data on notifiable infectious diseases in South Korea from 2016–2021 to examine how NPI implementation to control the COVID-19 pandemic affected patterns of various other diseases. We used data from 2016–2019 to develop a reliable time series model and then predicted the incidence of communicable diseases for 2020–2021 under the assumption that NPIs had not been implemented. By comparing the model-predicted values with observed values, we found that the incidence of respiratory infectious diseases decreased considerably after the implementation of NPIs. However, the incidence of human gastrointestinal infectious diseases and livestock diseases remained comparable or even increased after NPIs were implemented. The overall medical expenses associated with infectious diseases other than COVID-19 decreased by 5.17% in 2020 and 16.14% in 2021 compared with the predicted values (Table 3). Our findings offer valuable insights for implementing appropriate control measures during future epidemics.

The reductions in and the continuously low incidence levels of respiratory infectious diseases in South Korea during the COVID-19 pandemic can be attributed principally to the extensive adoption of NPIs. Regardless of whether the infectious agent was a bacterium (pertussis, scarlet fever, IPD, and TB) or a virus (varicella and mumps), respiratory infectious diseases transmitted via droplets, fomites, or direct contact generally exhibited lower incidence levels after implementation of NPIs; most of those trends persisted until the end of 2021. The sharp decline in the respiratory infectious disease incidence after implementation of NPIs was consistent with the findings of previous studies on the occurrence trends of respiratory infectious diseases in South Korea (*5*,*15*–*17*,*37*,*38*) and the findings of studies that focused on respiratory infectious disease patterns in other countries, such as China and the United States (*9*,*39*–*41*). 

After NPI implementation, the number of mumps cases remained lower than predicted. However, beginning in October 2021, the number of cases increased above the expected value. That change can be attributed to the nationwide relaxation of school attendance criteria in the fall semester of 2021, which led to more outbreaks in schools. Mumps is commonly observed among adolescents 13–18 years of age and frequently spreads in settings where persons engage in group activities (e.g., schools) (*42*). Therefore, precautions are needed to prevent a mumps resurgence after cessation of NPIs.

TB exhibited a slightly different pattern from those of other respiratory infectious diseases. In the early stages of NPI implementation, the number of cases noticeably decreased. However, beginning in the second half of 2020, we saw little or no difference between the observed and the predicted values. Reductions in TB notifications in early 2020, because of complex factors affecting disease diagnosis, have been reported in several countries, including South Korea (*19*,*43*). However, the effects of NPIs known to prevent acute infections were limited in suppressing TB cases in South Korea in the medium- to long-term because a many cases are presumed to arise when latent infections progress to active TB disease (*19*,*44*). Thus, even if NPIs are implemented, the existing strategies focusing on prophylactic treatment to prevent new infections and treating latent infections to prevent active TB onset still need to be incorporated.

The incidence of gastrointestinal diseases did not decrease after implementation of NPIs. Studies using data from countries such as China (*9*) and the United States (*45*) revealed notable decreases in most gastrointestinal infectious diseases after NPI implementation. Although the dissimilar contexts hinder direct comparisons, differences in the extent of NPIs and accessibility to medical services might explain the discrepancies. In the early stages of the COVID-19 pandemic, China and the United States put in place strict social distancing measures and emphasized stay-at-home orders. In contrast, South Korea used less strict policies that focused on personal hygiene measures. Therefore, the effectiveness of NPIs in terms of controlling infectious diseases might have varied among countries, and the decrease in healthcare facility utilization may have been smaller in South Korea (*18*,*19*,*45*). In addition, the gastrointestinal diseases included in this study were primarily foodborne diseases that commence after consumption of contaminated food or water (*20*). Therefore, the occurrence of the foodborne diseases included might not have been greatly affected by personal hygiene enhancement or social distancing measures.

This study revealed inconsistent temporal trends between the 2 target zoonotic diseases in industrial animals: bovine TB and brucellosis. The increased incidence of brucellosis was consistent with the prior predictions. Social distancing is likely to compromise appropriate veterinary care and restrict the logistical activities necessary for good livestock management (*11*). Moreover, in South Korea, the number of cattle farms increased during social distancing, possibly because of the increased profit to be made from beef (*46*). The sudden increases in disease incidence could indicate an increased number of inexperienced cattle owners, which would influence management quality. Because the primary route of brucellosis transmission is associated with the mass movement of infected cattle (*47*), inexperienced owners might need to require better brucellosis screening skills. However, accurate indicators of livestock movement during the COVID-19 period could not be collected. In contrast, the decreased incidence of bovine TB differed from our expectations. One possible explanation is that bovine TB surveillance increased in South Korea; the number of cattle screened for bovine tuberculosis infection has risen since 2017, as has the relevant budget (Appendix 1 Table 32) (*48*). Because early detection via effective surveillance plays a key role in controlling chronic diseases with long latent periods, the decreased incidence might be explained by effective surveillance efforts.

The first limitation of this study is that the incidence levels of infectious diseases are influenced by various factors, including population immunity, seasonal changes, climatic features, and human mobility patterns. Thus, drawing causal inferences regarding the effects of social distancing measures and changes on disease patterns is challenging. We can only interpret and analyze potential influencing factors. Second, the observed decreases in the incidence of certain infectious diseases might not solely reflect the effects of NPIs on incidence rates. The decreases also could be influenced by other pandemic-related factors, including healthcare utilization. Thus, we examined annual hospital visits and health insurance claims to adjust for any changes in healthcare utilization. However, biases might have persisted in terms of altered healthcare-seeking behaviors and surveillance capacities. Moreover, given the strict infection control regulations, healthcare utilization by symptomatic patients was particularly restricted. Therefore, the data on healthcare utilization among all patients considered in this study might not fully reflect the reduction in healthcare use by those with symptoms. Third, although ARIMA is a well-established and practical technology for infectious disease forecasting (*22*,*41*), the method has limitations in distinguishing various factors that affect transmission, such as genetic strain and latent infections. Furthermore, ARIMA might not be the most appropriate method for long-term predictions. However, the infectious diseases targeted in this study generally exhibit stable trends, with clear seasonal variabilities, and the fitted models indeed exhibited relatively good fits with the training data and reasonably good predictive performances, as confirmed by out-of-sample validation. In addition, the time-series forecasting models were used in previous studies to predict influenza virus activity for 2020–2022 (*49*), or to estimate excess mortality during the COVID-19 period, 2020–2021 (*50*). Therefore, we believe that the reliability of long-term predictions of the incidence of the chosen diseases remains robust. Thus, we used the ARIMA approach (a descriptive method) to present our results. Finally, this study did not consider demographic information, such as age and sex.

In conclusion, the implementation of NPIs considerably reduced the incidence of infectious diseases transmitted via respiratory routes or direct person-to-person contact in South Korea, a trend that continued until late 2021. Although identifying a single factor that explains changes in the incidence of all infectious diseases is difficult, the concurrent implementation of NPIs at various levels (individual, community, environmental, and national), along with behavioral changes, likely played a key role in reducing community transmission and alleviating the associated healthcare burden. Therefore, comprehensive NPI strategies are critical public health considerations for controlling infectious diseases and preparing for future pandemics.

Appendix 1 Additional tables and figures for study of trends in nationally notifiable infectious diseases in humans and animals during the COVID-19 pandemic, South Korea.

Appendix 2 Additional data for study of trends in nationally notifiable infectious diseases in humans and animals during the COVID-19 pandemic, South Korea.

## References

[1] Wang H, Paulson KR, Pease SA, Watson S, Comfort H, Zheng P, et al.; COVID-19 Excess Mortality Collaborators. Estimating excess mortality due to the COVID-19 pandemic: a systematic analysis of COVID-19-related mortality, 2020-21. Lancet. 2022;399:1513–36. 10.1016/S0140-6736(21)02796-3 35279232 PMC8912932

[2] Bielecki M, Züst R, Siegrist D, Meyerhofer D, Crameri GAG, Stanga Z, et al. Social distancing alters the clinical course of COVID-19 in young adults: a comparative cohort study. Clin Infect Dis. 2021;72:598–603. 10.1093/cid/ciaa889 32594121 PMC7337655

[3] Giles ML, Wallace EM, Alpren C, Brady N, Crouch S, Romanes F, et al. Suppression of severe acute respiratory syndrome coronavirus 2 (SARS-CoV-2) after a second wave in Victoria, Australia. Clin Infect Dis. 2021;73:e808–10. 10.1093/cid/ciaa1882 33354719 PMC7799206

[4] Tsai AC, Harling G, Reynolds Z, Gilbert RF, Siedner MJ. Coronavirus disease 2019 (COVID-19) transmission in the United States before versus after relaxation of statewide social distancing measures. Clin Infect Dis. 2021;73(Suppl 2):S120–6. 10.1093/cid/ciaa1502 33009800 PMC7797755

[5] Ahn JG. Epidemiological changes in infectious diseases during the coronavirus disease 2019 pandemic in Korea: a systematic review. Clin Exp Pediatr. 2022;65:167–71. 10.3345/cep.2021.01515 34844396 PMC8990948

[6] Min KD, Kang H, Lee JY, Jeon S, Cho SI. Estimating the effectiveness of non-pharmaceutical interventions on COVID-19 control in Korea. J Korean Med Sci. 2020;35:e321. 10.3346/jkms.2020.35.e321 32893522 PMC7476801

[7] Choi JH. Effects of nonpharmaceutical interventions for coronavirus disease 2019. Clin Exp Pediatr. 2022;65:250–1. 10.3345/cep.2021.01830 35344981 PMC9082245

[8] Chow EJ, Uyeki TM, Chu HY. The effects of the COVID-19 pandemic on community respiratory virus activity. Nat Rev Microbiol. 2023;21:195–210. 36253478 10.1038/s41579-022-00807-9 PMC9574826

[9] Geng MJ, Zhang HY, Yu LJ, Lv CL, Wang T, Che TL, et al. Changes in notifiable infectious disease incidence in China during the COVID-19 pandemic. Nat Commun. 2021;12:6923. 10.1038/s41467-021-27292-7 34836947 PMC8626444

[10] Ahn SY, Park JY, Lim IS, Chae SA, Yun SW, Lee NM, et al. Changes in the occurrence of gastrointestinal infections after COVID-19 in Korea. J Korean Med Sci. 2021;36:e180. 10.3346/jkms.2021.36.e180 34155841 PMC8216988

[11] Rahman MT, Islam MS, Shehata AA, Basiouni S, Hafez HM, Azhar EI, et al. Influence of COVID-19 on the sustainability of livestock performance and welfare on a global scale. Trop Anim Health Prod. 2022;54:309. 10.1007/s11250-022-03256-x 36114917 PMC9483476

[12] Hashem NM, González-Bulnes A, Rodriguez-Morales AJ. Animal welfare and livestock supply chain sustainability under the COVID-19 outbreak: an overview. Front Vet Sci. 2020;7:582528. 10.3389/fvets.2020.582528 33195601 PMC7593325

[13] Raihan A, Himu HA. Global impact of COVID-19 on the sustainability of livestock production. Global Sustainability Research. 2023;2:1–11. 10.56556/gssr.v2i2.447

[14] Huh K, Kim YE, Ji W, Kim DW, Lee EJ, Kim JH, et al. Decrease in hospital admissions for respiratory diseases during the COVID-19 pandemic: a nationwide claims study. Thorax. 2021;76:939–41. 10.1136/thoraxjnl-2020-216526 33782081

[15] Kim JH, Roh YH, Ahn JG, Kim MY, Huh K, Jung J, et al. Respiratory syncytial virus and influenza epidemics disappearance in Korea during the 2020-2021 season of COVID-19. Int J Infect Dis. 2021;110:29–35. 10.1016/j.ijid.2021.07.005 34245886

[16] Lee H, Lee H, Song K-H, Kim ES, Park JS, Jung J, et al. Impact of public health interventions on seasonal influenza activity during the COVID-19 outbreak in Korea. Clin Infect Dis. 2021;73:e132–40. 10.1093/cid/ciaa672 32472687 PMC7314207

[17] Park S, Michelow IC, Choe YJ. Shifting patterns of respiratory virus activity following social distancing measures for coronavirus disease 2019 in South Korea. J Infect Dis. 2021;224:1900–6. 10.1093/infdis/jiab231 34009376 PMC8135809

[18] Yun HE, Ryu BY, Choe YJ. Impact of social distancing on incidence of vaccine-preventable diseases, South Korea. J Med Virol. 2021;93:1814–6. 10.1002/jmv.26614 33079384

[19] Kwak N, Hwang S-S, Yim J-J. Effect of COVID-19 on tuberculosis notification, South Korea. Emerg Infect Dis. 2020;26:2506–8. 10.3201/eid2610.202782 32672531 PMC7510739

[20] Park S, Michelow IC, Choe YJ. Trend of gastrointestinal infections following nonpharmaceutical interventions, South Korea, 2020. J Infect Dis. 2021;224:368–71. 10.1093/infdis/jiab244 33963753

[21] Scallan E, Mahon BE, Hoekstra RM, Griffin PM. Estimates of illnesses, hospitalizations and deaths caused by major bacterial enteric pathogens in young children in the United States. Pediatr Infect Dis J. 2013;32:217–21. 10.1097/INF.0b013e31827ca763 23249909

[22] Benvenuto D, Giovanetti M, Vassallo L, Angeletti S, Ciccozzi M. Application of the ARIMA model on the COVID-2019 epidemic dataset. Data Brief. 2020;29:105340. 10.1016/j.dib.2020.105340 32181302 PMC7063124

[23] Cori A, Ferguson NM, Fraser C, Cauchemez S. A new framework and software to estimate time-varying reproduction numbers during epidemics. Am J Epidemiol. 2013;178:1505–12. 10.1093/aje/kwt133 24043437 PMC3816335

[24] National Health Insurance Service. Status of medical treatment by region 2006–2021 [in Korean] [cited 2023 Jan 11]. https://kosis.kr/statHtml/statHtml.do?orgId=350&tblId=TX_35003_A004&vw_cd=MT_ZTITLE&list_id=350_35003_1&scrId=&seqNo=&lang_mode=ko&obj_var_id=&itm_id=&conn_path=MT_ZTITLE&path=%252FstatisticsList%252FstatisticsListIndex.do

[25] Health Insurance Review and Assessment Service. Total medical expenses status [in Korean] [cited 2023 Jan 11]. http://opendata.hira.or.kr/op/opc/olapHthInsRvStatInfo.do

[26] Korea Statistics. Total population of South Korea [in Korean] [cited 2023 Jan 13]. https://kosis.kr/visual/populationKorea/PopulationDashBoardDetail.do

[27] Health Insurance Review and Assessment Service. Infectious disease treatment expenses statistics [in Korean] [cited 2023 Jan 14]. https://www.data.go.kr/data/15085952/fileData.do

[28] Song JY, Peck KR; Korean Society of Infectious Diseases. A debate on public health responses to COVID-19: focused protection versus sustained suppression. J Korean Med Sci. 2020;35:e433–0. 10.3346/jkms.2020.35.e433 33350189 PMC7752261

[29] Central Disaster and Safety Countermeasure Headquarters of the Republic of Korea. Rules and guidelines for distancing in daily life to control coronavirus disease 2019 in Korea: 3rd version, announced on July 3, 2020. J Educ Eval Health Prof. 2020;17:20. 10.3352/jeehp.2020.17.20 32663925 PMC7403533

[30] Korea Disease Control and Prevention Agency. Infectious disease portal. 2023 [in Korean] [cited 2023 Jan 21]. https://www.mohw.go.kr/react/al/sal0301vw.jsp

[31] Animal and Plant Quarantine Agency. Korea Animal Health integrated system [in Korean] [cited 2023 Jan 21]. https://home.kahis.go.kr/home/lkntscrinfo/selectLkntsOccrrncList.do

[32] Ministry of Agriculture. Food and Rural Affairs. Regional livestock farming status [in Korean] [cited 2023 Jan 26]. https://uni.agrix.go.kr/docs7/biOlap/fixType.do

[33] Campbell F, Strang C, Ferguson N, Cori A, Jombart T. When are pathogen genome sequences informative of transmission events? PLoS Pathog. 2018;14:e1006885. 10.1371/journal.ppat.1006885 29420641 PMC5821398

[34] Lau EHY, Nishiura H, Cowling BJ, Ip DKM, Wu JT. Scarlet fever outbreak, Hong Kong, 2011. Emerg Infect Dis. 2012;18:1700–2. 10.3201/eid1810.120062 23017843 PMC3471616

[35] Vink MA, Bootsma MCJ, Wallinga J. Serial intervals of respiratory infectious diseases: a systematic review and analysis. Am J Epidemiol. 2014;180:865–75. 10.1093/aje/kwu209 25294601

[36] Dritsakis N, Klazoglou P. Forecasting unemployment rates in USA using Box-Jenkins methodology. Int J Econ Financial Issues. 2018;8:9–20.

[37] Shi HJ, Kim NY, Eom SA, Kim-Jeon MD, Oh SS, Moon BS, et al. Effects of non-pharmacological interventions on respiratory viruses other than SARS-CoV-2: analysis of laboratory surveillance and literature review from 2018 to 2021. J Korean Med Sci. 2022;37:e172. 10.3346/jkms.2022.37.e172 35638198 PMC9151990

[38] Yum S, Hong K, Sohn S, Kim J, Chun BC. Trends in viral respiratory infections during COVID-19 pandemic, South Korea. Emerg Infect Dis. 2021;27:1685–8. 10.3201/eid2706.210135 34013875 PMC8153859

[39] Li ZJ, Yu LJ, Zhang HY, Shan CX, Lu QB, Zhang XA, et al.; Chinese Centers for Disease Control and Prevention (CDC) Etiology Surveillance Study Team of Acute Respiratory Infections. Broad impacts of coronavirus disease 2019 (COVID-19) pandemic on acute respiratory infections in China: an observational study. Clin Infect Dis. 2022;75:e1054–62. 10.1093/cid/ciab942 34788811 PMC8767888

[40] Fricke LM, Glöckner S, Dreier M, Lange B. Impact of non-pharmaceutical interventions targeted at COVID-19 pandemic on influenza burden - a systematic review. J Infect. 2021;82:1–35. 10.1016/j.jinf.2020.11.039 33278399 PMC9183207

[41] Feng L, Zhang T, Wang Q, Xie Y, Peng Z, Zheng J, et al. Impact of COVID-19 outbreaks and interventions on influenza in China and the United States. Nat Commun. 2021;12:3249. 10.1038/s41467-021-23440-1 34059675 PMC8167168

[42] Park SH. Resurgence of mumps in Korea. Infect Chemother. 2015;47:1–11. 10.3947/ic.2015.47.1.1 25844258 PMC4384450

[43] Cilloni L, Fu H, Vesga JF, Dowdy D, Pretorius C, Ahmedov S, et al. The potential impact of the COVID-19 pandemic on the tuberculosis epidemic a modelling analysis. EClinicalMedicine. 2020;28:100603. 10.1016/j.eclinm.2020.100603 33134905 PMC7584493

[44] Jeong D, Kang HY, Kim J, Lee H, Yoo BN, Kim HS, et al. Cohort rpofile: Korean tuberculosis and post-tuberculosis cohort constructed by linking the Korean National Tuberculosis Surveillance System and National Health Information Database. J Prev Med Public Health. 2022;55:253–62. 10.3961/jpmph.21.635 35677999 PMC9201096

[45] Kim S, Kim J, Choi BY, Park B. Trends in gastrointestinal infections before and during non-pharmaceutical interventions in Korea in comparison with the United States. Epidemiol Health. 2022;44:e2022011. 10.4178/epih.e2022011 34990526 PMC9117109

[46] Lee HW, Ji SW, Lee Y, Kim HJ, Song WJ. Recent reasons for the decline in Hanwoo (Korean native cattle) prices and outlook [in Korean]. Naju; Republic of Korea: Korea Rural Economic Institute; 2022.

[47] Yoon H, Moon O-K, Lee S-H, Lee W-C, Her M, Jeong W, et al. Epidemiology of brucellosis among cattle in Korea from 2001 to 2011. J Vet Sci. 2014;15:537–43. 10.4142/jvs.2014.15.4.537 25234321 PMC4269596

[48] Ministry of Agriculture. Food and Rural Affairs. Guidelines for livestock epidemic prevention and control, 2022 [in Korean] [cited 2023 Jan 21]. https://www.mafra.go.kr/bbs/mafra/71/419654/artclView.do

[49] Kim HK, Min KD, Cho SI. Analysis of the effectiveness of non-pharmaceutical interventions on influenza during the Coronavirus disease 2019 pandemic by time-series forecasting. BMC Infect Dis. 2023;23:717. 10.1186/s12879-023-08640-y 37875817 PMC10594831

[50] Wang H, Paulson KR, Pease SA, Watson S, Comfort H, Zheng P, et al.; COVID-19 Excess Mortality Collaborators. Estimating excess mortality due to the COVID-19 pandemic: a systematic analysis of COVID-19-related mortality, 2020-21. Lancet. 2022;399:1513–36. 10.1016/S0140-6736(21)02796-3 35279232 PMC8912932

