# Supplementary material for: Trends in Nationally Notifiable Infectious Diseases in Humans and Animals during COVID-19 Pandemic, South Korea
Source: Emerg Infect Dis. 2024 Jun;30(6):1154–63. doi: 10.3201/eid3006.231422 (PMC11138988; doi:10.3201/eid3006.231422)
Supplement: Appendix 1 — Additional tables and figures for study of trends in nationally notifiable infectious diseases in humans and animals during the COVID-19 pandemic, South Korea. [file 23-1422-Techapp-s1.pdf]

Article DOI: http://doi.org/10.3201/eid3006.231422

EID cannot ensure accessibility for supplementary materials supplied by authors. Readers who have difficulty accessing supplementary content should contact the authors for assistance.

Trends in Nationally Notifiable Infectious Diseases in Humans and Animals during COVID-19 Pandemic, South Korea

Appendix 1

Appendix 1 Table 1. The information on annual hospital visits and annual health insurance claims in Korea.

Year	Annual hospital visits	Annual health insurance claims
2010	46,690,017	1,255,867,542
2011	47,354,032	1,280,066,253
2012	47,468,197	1,363,271,451
2013	47,756,770	1,368,207,434
2014	48,278,590	1,393,912,709
2015	48,560,635	1,389,266,897
2016	49,011,735	1,434,276,197
2017	49,173,457	1,452,720,186
2018	49,472,060	1,475,254,622
2019	49,634,160	1,516,941,079
2020	48,570,460	1,321,183,845
2021	49,140,278	1,306,717,932

Appendix 1 Table 2. Three-tier Social Distancing System in Response to Coronavirus Disease 2019 Outbreak, South Korea (June 28, 2020 to November 2020)

Social Distancing	Level 1	Level 2	Level 3
The general terms	Distancing in Daily Life	Social Distancing, Moderate Distancing	Intensive Social Distancing
The average number of confirmed cases over a period of two weeks (excluding imported cases)	If the average number of confirmed cases is 50 or less	If the number is between 50 and 100 with a regional surge	If the number is 100 or more and doubles at least twice in one week or more
Key Messages	Adherence to hygiene guidelines, allowing for normal economic activities	Avoiding unnecessary outings, gatherings, and use of crowded facilities	Essential social and economic activities should be prioritized, while all other activities should be avoided

Appendix 1 Table 3. Five-tier Social Distancing System in Response to Coronavirus Disease 2019 Outbreak, South Korea (November 2020 to July 2021)

<table>
<tr><th>Social Distancing</th><th>Level 1</th><th>Level 1.5</th><th>Level 2</th><th>Level 2.5</th><th>Level 3</th></tr>
<tr><td>The general terms</td><td>Distancing in Daily Life</td><td>Local Outbreak Initiation</td><td>Rapid Local Spread, Nationwide Spread Initiation</td><td>Nationwide Outbreak Intensification</td><td>Nationwide Major Epidemic</td></tr>
<tr><td>Condition of medical system</td><td>Routine Management</td><td>Localized Threat</td><td>Difficulty in Local Response</td><td>Exceeding Nationwide Response Capacity</td><td>Risk of Healthcare System Collapse</td></tr>
<tr><td>Exhibitions, Trade Shows, International Conferences</td><td rowspan="2">Consultation with local authorities required with 500 or more participants</td><td rowspan="2">Prohibited with 100 or more participants</td><td>1 person per 4m^2 occupancy limit</td><td>1 person per 16m^2 occupancy limit</td><td rowspan="2">Prohibited if there are 10 or more participants</td></tr>
<tr><td>Other Gatherings, Meetings, Events</td><td>Prohibited with 100 or more participants</td><td>Prohibited with 50 or more participants</td></tr>
</table>

Appendix 1 Table 4. Four-tier Social Distancing System in Response to Coronavirus Disease 2019 Outbreak, South Korea July 2021 to onward)

Social Distancing	Level 1	Level 2	Level 3	Level 4
The general terms	Sustained Suppression Phase	Regional Outbreak	Regional Epidemic	Nationwide Epidemic
Condition of medical system	Conventional medical response	Limited regional infection control	Limited regional infection control (intensified)	Limited nationwide infection control
Overview of Response	Adherence to facility-specific and individual-level hygiene measures to prevent crowding, confined spaces, and close contact.	Restriction on the number of people allowed in a facility or venue.	Prohibition of private gatherings or social events.	Avoidance of unnecessary outings or travel, Encouragement to stay at home and minimize outdoor activities.
Criteria: cases per week per 100,000 population	Less than 1 case	1 or more cases	2 or more cases	4 or more cases

Appendix 1 Table 5. The list of target diseases.

Disease category	Number	Disease name	Temporal range	Temporal resolution	Modeling outcome
Respiratory	1	Varicella	2016~2021	Weekly	Cases
	2	Pertussis	2016~2021		(Calculated from
	3	Mumps	2016~2021		R_t)
	4	Invasive pneumococcal disease	2016~2021		
	5	Scarlet fever	2016~2021		
	6	Tuberculosis	2016~2021	Monthly	Cases
Gastrointestinal	1	Thyphoid fever	2016~2021	Monthly	Cases
	2	Shigellosis	2016~2021		
	3	Hepatitis A	2016~2021		
	4	Enterohemorrhagic *Escherichia coli*	2016~2021		
Livestock diseases (zoonotic)	1	Cattle tuberculosis	2016~2021	Monthly	Cases
	2	Cattle brucellosis	2016~2021		

Appendix 1 Table 6. Serial intervals of respiratory diseases and their standard deviations.

Respiratory disease	Serial interval (days)	Standard deviation (days)
Varicella	14.0	2.2
Pertussis	22.8	6.5
Mumps	18.0	3.5
Invasive pneumococcal diseases (*Streptococcus pneumoniae*)	6.6	1.8
Scarlet fever	14.0	4.9

Appendix 1 Table 7. The differencing applied to each infectious disease data and the stationarity of the data after differencing.

No.	Diseases name	Applied First Differencing	Applied Seasonal Differencing	ADF (Augmented Dickey-Fuller) Test	KPSS (Kwiatkowski-Phillips-Schmidt-Shin) Test
1	Varicella	0	1	<0.01	0.46
2	Pertussis	1	1	0.06	0.08
3	Mumps	0	1	0.08	0.12
4	Invasive pneumococcal disease	0	1	<0.01	0.27
5	Scarlet fever	1	1	<0.01	0.24
6	Tuberculosis	1	0	<0.01	0.18
7	Thyphoid fever	1	0	<0.01	0.12
8	Shigellosis	1	1	<0.05	0.32
9	Hepatitis A	1	0	0.07	0.11
10	Enterohemorrhagic *E. coli*	0	1	<0.05	0.11
11	Cattle tuberculosis	0	0	0.13	0.19
12	Cattle brucellosis	0	0	<0.05	0.53

Appendix 1 Table 8. Parameters and diagnostic statistics of candidate ARIMA models of Varicella incidences.

Model	AICc	AIC	BIC	RMSE
ARIMA(3,0,2)(0,1,0)[52]	−618.75	−619.34	−601.23	0.03
ARIMA(3,0,1)(0,1,0)[52]	−568.56	−568.98	−553.89	0.03
ARIMA(2,0,2)(0,1,0)[52]	−567.91	−568.32	−553.23	0.04
ARIMA(3,1,2)(0,1,0)[52]	−616.92	−617.51	−599.44	0.03
ARIMA(3,0,2)(1,1,0)[52]	−607.72	−608.5	−587.38	0.03
ARIMA(3,0,2)(0,1,1)[52]	−591.79	−592.57	−571.45	0.03
ARIMA(4,0,2)(0,1,0)[52]	−586.47	−587.25	−566.13	0.04
ARIMA(3,0,3)(0,1,0)[52]	−579.71	−580.49	−559.37	0.04
ARIMA(4,0,3)(0,1,0)[52]	−588.23	−589.24	−565.11	0.04
ARIMA(4,1,3)(0,1,0)[52]	−612.51	−613.53	−589.44	0.03

Appendix 1 Table 9. Diagnostic statistics of candidate ARIMA models of Varicella incidences, assessed with out-of-sample validation.

Model	MAE	RMSE	Theil's U
ARIMA(3,0,2)(0,1,0)[52]	0.22	0.27	0.21
ARIMA(3,0,1)(0,1,0)[52]	0.36	0.54	0.44
ARIMA(2,0,2)(0,1,0)[52]	0.24	0.31	0.24
ARIMA(3,1,2)(0,1,0)[52]	0.21	0.28	0.22
ARIMA(3,0,2)(1,1,0)[52]	0.31	0.39	0.31
ARIMA(3,0,2)(0,1,1)[52]	0.28	0.38	0.29
ARIMA(4,0,2)(0,1,0)[52]	0.27	0.35	0.22
ARIMA(3,0,3)(0,1,0)[52]	0.28	0.36	0.29
ARIMA(4,0,3)(0,1,0)[52]	0.28	0.36	0.29
ARIMA(4,1,3)(0,1,0)[52]	0.26	0.38	0.31

Appendix 1 Table 10. Parameters and diagnostic statistics of candidate ARIMA models of Pertussis incidences.

Model	AICc	AIC	BIC	RMSE
ARIMA(4,1,0)(1,1,0)[52]	−560.81	−561.45	−543.80	0.05
ARIMA(4,1,0)(1,0,0)[52]	−868.08	−868.53	−848.99	0.03
ARIMA(4,1,0)(0,0,1)[52]	−868.14	−868.60	−849.05	0.03
ARIMA(4,1,0)(1,0,1)[52]	−868.15	−868.76	−845.96	0.03
ARIMA(4,1,1)(1,1,1)[52]	−866.38	−867.17	−841.11	0.03
ARIMA(5,1,0)(1,0,1)[52]	−867.09	−867.88	−841.82	0.03
ARIMA(3,1,0)(1,0,1)[52]	−852.7	−853.15	−833.61	0.04
ARIMA(4,1,2)(1,0,1)[52]	−877.79	−878.77	−849.46	0.04
ARIMA(4,0,3)(0,1,0)[52]	−588.23	−589.24	−565.11	0.03
ARIMA(4,1,3)(0,1,0)[52]	−612.51	−613.53	−589.44	0.03

Appendix 1 Table 11. Diagnostic statistics of candidate ARIMA models of Pertussis incidences, assessed with out-of-sample validation.

Model	MAE	RMSE	Theil's U
ARIMA(4,1,0)(1,1,0)[52]	0.45	0.66	0.71
ARIMA(4,1,0)(1,0,0)[52]	0.38	0.58	0.61
ARIMA(4,1,0)(0,0,1)[52]	0.49	0.63	0.67
ARIMA(4,1,0)(1,0,1)[52]	0.49	0.63	0.67
ARIMA(4,1,1)(1,1,1)[52]	0.37	0.58	0.62
ARIMA(5,1,0)(1,0,1)[52]	0.51	0.64	0.68
ARIMA(3,1,0)(1,0,1)[52]	0.69	0.84	0.89
ARIMA(4,1,2)(1,0,1)[52]	0.49	0.63	0.67
ARIMA(4,0,3)(0,1,0)[52]	0.86	1.17	1.24
ARIMA(4,1,3)(0,1,0)[52]	0.49	0.63	0.67

Appendix 1 Table 12. Parameters and diagnostic statistics of candidate ARIMA models of Mumps incidences.

Model	AICc	AIC	BIC	RMSE
ARIMA(1,2,2)(1,1,0)[52]	−702.98	−703.41	−688.57	0.03
ARIMA(1,0,2)(1,1,0)[52]	−788.12	−788.55	−773.63	0.02
ARIMA(1,0,2)(1,1,1)[52]	−786.45	−787.06	−769.15	0.02
ARIMA(2,0,2)(1,1,0)[52]	−774.36	−774.96	−757.06	0.03
ARIMA(1,0,1)(1,1,0)[52]	−766.38	−767.17	−741.11	0.02
ARIMA(1,0,1)(0,1,0)[52]	−643.13	−643.3	−634.35	0.03
ARIMA(2,0,1)(1,1,0)[52]	−711.38	−711.81	−696.89	0.04
ARIMA(0,0,1)(1,1,0)[52]	−823.79	−823.32	−814.46	0.02
ARIMA(1,1,1)(1,1,0)[52]	−709.23	−689.24	−665.11	0.03
ARIMA(2,1,1)(1,1,0)[52]	−784.51	−785.53	−770.44	0.03

Appendix 1 Table 13. Diagnostic statistics of candidate ARIMA models of Mumps incidences, assessed with out-of-sample validation.

Model	MAE	RMSE	Theil's U
ARIMA(1,2,2)(1,1,0)[52]	0.04	0.06	0.05
ARIMA(1,0,2)(1,1,0)[52]	0.06	0.07	0.05
ARIMA(1,0,2)(1,1,1)[52]	0.06	0.06	0.05
ARIMA(2,0,2)(1,1,0)[52]	0.19	0.27	0.22
ARIMA(1,0,1)(1,1,0)[52]	0.05	0.06	0.05
ARIMA(1,0,1)(0,1,0)[52]	0.05	0.07	0.05
ARIMA(2,0,1)(1,1,0)[52]	0.31	0.38	0.31
ARIMA(0,0,1)(1,1,0)[52]	0.05	0.06	0.05
ARIMA(1,1,1)(1,1,0)[52]	0.54	0.61	0.48
ARIMA(2,1,1)(1,1,0)[52]	0.54	0.61	0.48

Appendix 1 Table 14. Parameters and diagnostic statistics of candidate ARIMA models of invasive pneumococcal disease incidences.

Model	AICc	AIC	BIC	RMSE
ARIMA(2,0,0)(1,1,1)[52]	−570.40	−570.97	−552.67	0.04
ARIMA(2,0,1)(1,1,1)[52]	−567.45	−568.01	−549.71	0.04
ARIMA(1,0,0)(1,1,1)[52]	−334.94	−335.2	−323.00	0.05
ARIMA(1,0,1)(1,1,1)[52]	−428.78	−429.18	−413.93	0.04
ARIMA(3,0,0)(1,1,1)[52]	−567.59	−568.16	−549.86	0.04
ARIMA(3,0,1)(1,1,1)[52]	−570.55	−571.31	−559.96	0.03
ARIMA(2,0,0)(1,1,0)[52]	−570.50	−570.76	−558.56	0.04
ARIMA(2,0,0)(0,1,0)[52]	−507.47	−507.63	−498.48	0.04
ARIMA(2,0,0)(0,1,1)[52]	−557.43	−557.7	−545.5	0.04
ARIMA(2,0,0)(1,0,1)[52]	−484.51	−485.53	−470.44	0.03

Appendix 1 Table 15. Diagnostic statistics of candidate ARIMA models of invasive pneumococcal disease incidences, assessed with out-of-sample validation.

Model	MAE	RMSE	Theil's U
ARIMA(2,0,0)(1,1,1)[52]	0.65	0.91	0.81
ARIMA(2,0,1)(1,1,1)[52]	0.28	0.32	0.29
ARIMA(1,0,0)(1,1,1)[52]	0.26	0.32	0.29
ARIMA(1,0,1)(1,1,1)[52]	0.28	0.41	0.36
ARIMA(3,0,0)(1,1,1)[52]	0.26	0.32	0.28
ARIMA(3,0,1)(1,1,1)[52]	0.61	0.65	0.58
ARIMA(2,0,0)(1,1,0)[52]	0.35	0.48	0.51
ARIMA(2,0,0)(0,1,0)[52]	0.28	0.34	0.31
ARIMA(2,0,0)(0,1,1)[52]	0.79	1.12	0.84
ARIMA(2,0,0)(1,0,1)[52]	0.92	1.18	0.94

Appendix 1 Table 16. Parameters and diagnostic statistics of candidate ARIMA models of scarlet fever incidences.

Model	AICc	AIC	BIC	RMSE
ARIMA(2,1,3)(0,1,0)[52]	−954.93	−955.53	−937.55	0.01
ARIMA(2,1,2)(0,1,0)[52]	−952.57	−953.00	−938.71	0.01
ARIMA(3,1,2)(0,1,0)[52]	−926.83	−927.42	−909.44	0.01
ARIMA(1,1,2)(0,1,0)[52]	−898.41	−898.69	−886.70	0.01
ARIMA(2,1,1)(0,1,0)[52]	−946.91	−947.19	−935.20	0.01
ARIMA(2,1,2)(1,1,0)[52]	−948.49	−949.09	−931.11	0.01
ARIMA(2,1,2)(1,1,1)[52]	−947.69	−948.49	−947.51	0.01
ARIMA(2,1,2)(2,1,0)[52]	−855.98	−856.78	−835.80	0.01
ARIMA(2,1,2)(0,1,1)[52]	−956.58	−957.17	−939.19	0.01
ARIMA(2,1,2)(2,1,1)[52]	−898.62	−899.65	−875.67	0.01

Appendix 1 Table 17. Diagnostic statistics of candidate ARIMA models of scarlet fever incidences, assessed with out-of-sample validation.

Model	MAE	RMSE	Theil's U
ARIMA(2,1,3)(0,1,0)[52]	0.42	0.44	0.39
ARIMA(2,1,2)(0,1,0)[52]	0.39	0.42	0.38
ARIMA(3,1,2)(0,1,0)[52]	0.42	0.45	0.41
ARIMA(1,1,2)(0,1,0)[52]	0.64	0.68	0.65
ARIMA(2,1,1)(0,1,0)[52]	0.24	0.26	0.25
ARIMA(2,1,2)(1,1,0)[52]	0.69	0.28	0.71
ARIMA(2,1,2)(1,1,1)[52]	0.73	0.73	0.37
ARIMA(2,1,2)(2,1,0)[52]	0.39	0.78	0.76
ARIMA(2,1,2)(0,1,1)[52]	0.22	0.26	0.23
ARIMA(2,1,2)(2,1,1)[52]	0.73	0.78	0.72

Appendix 1 Table 18. Parameters and diagnostic statistics of candidate ARIMA models of Tuberculosis incidences.

Model	AICc	AIC	BIC	RMSE
ARIMA(0,1,3)(1,0,0)[12]	−158.37	−159.76	−150.32	21.87
ARIMA(1,1,3)(1,0,0)[12]	−157.84	−159.84	−148.49	21.27
ARIMA(1,1,4)(1,0,0)[12]	−157.34	−160.07	−146.83	19.25
ARIMA(0,1,4)(1,0,0)[12]	−159.54	−161.54	−150.19	20.21
ARIMA(0,1,4)(1,0,1)[12]	−156.94	−159.68	−146.43	20.05
ARIMA(0,1,4)(2,0,0)[12]	−156.87	−159.61	−146.36	20.13
ARIMA(0,1,4)(2,0,1)[12]	−154.22	−157.82	−146.53	19.92
ARIMA(0,1,5)(1,0,0)[12]	−157.04	−159.78	−146.53	20.19
ARIMA(0,1,2)(1,0,0)[12]	−149.13	−150.04	−142.48	24.78
ARIMA(1,1,2)(1,0,0)[12]	−149.96	−151.35	−141.89	24.01

Appendix 1 Table 19. Diagnostic statistics of candidate ARIMA models of Tuberculosis incidences, assessed with out-of-sample validation.

Model	MAE	RMSE	Theil's U
ARIMA(0,1,3)(1,0,0)[12]	537.18	744.30	0.66
ARIMA(1,1,3)(1,0,0)[12]	536.38	744.08	0.67
ARIMA(1,1,4)(1,0,0)[12]	536.81	744.63	0.66
ARIMA(0,1,4)(1,0,0)[12]	536.34	744.11	0.69
ARIMA(0,1,4)(1,0,1)[12]	536.21	745.21	0.68
ARIMA(0,1,4)(2,0,0)[12]	536.42	743.13	0.66
ARIMA(0,1,4)(2,0,1)[12]	534.02	741.01	0.64
ARIMA(0,1,5)(1,0,0)[12]	537.52	743.96	0.66
ARIMA(0,1,2)(1,0,0)[12]	535.91	746.36	0.65
ARIMA(1,1,2)(1,0,0)[12]	536.17	742.01	0.65

Appendix 1 Table 20. Parameters and diagnostic statistics of candidate ARIMA models of Typhoid incidences.

Model	AICc	AIC	BIC	RMSE
ARIMA(0,1,3)(1,0,0)[12]	22.75	21.35	30.81	0.35
ARIMA(0,1,2)(1,0,0)[12]	33.85	32.94	40.51	0.42
ARIMA(0,1,4)(1,0,0)[12]	25.05	23.05	34.41	0.35
ARIMA(0,1,1)(1,0,0)[12]	31.54	31.01	36.68	0.42
ARIMA(1,1,3)(1,0,0)[12]	25.19	23.19	34.54	0.35
ARIMA(2,1,3)(1,0,0)[12]	24.31	21.57	34.82	0.34
ARIMA(0,1,3)(1,1,0)[12]	40.07	38.13	46.19	0.42
ARIMA(0,1,3)(1,1,1)[12]	40.06	37.26	46.93	0.31
ARIMA(0,1,3)(2,0,0)[12]	25.28	23.28	34.64	0.35
ARIMA(0,1,3)(2,0,1)[12]	27.49	24.75	38.01	0.34

Appendix 1 Table 21. Diagnostic statistics of candidate ARIMA models of Typhoid incidences, assessed with out-of-sample validation.

Model	MAE	RMSE	Theil's U
ARIMA(0,1,3)(1,0,0)[12]	1.02	2.07	1.42
ARIMA(0,1,2)(1,0,0)[12]	1.09	2.14	1.46
ARIMA(0,1,4)(1,0,0)[12]	1.11	2.11	1.45
ARIMA(0,1,1)(1,0,0)[12]	1.02	2.11	1.45
ARIMA(1,1,3)(1,0,0)[12]	1.09	2.12	1.45
ARIMA(2,1,3)(1,0,0)[12]	1.08	2.07	1.49
ARIMA(0,1,3)(1,1,0)[12]	1.16	2.24	1.49
ARIMA(0,1,3)(1,1,1)[12]	1.16	2.24	1.44
ARIMA(0,1,3)(2,0,0)[12]	1.07	2.08	1.47
ARIMA(0,1,3)(2,0,1)[12]	1.11	2.17	1.47

Appendix 1 Table 22. Parameters and diagnostic statistics of candidate ARIMA models of Shigellosis incidences.

Model	AICc	AIC	BIC	RMSE
ARIMA(3,1,0)(0,1,0)[12]	32.92	31.67	38.11	0.17
ARIMA(3,1,1)(0,1,0)[12]	34.88	32.94	41.02	0.16
ARIMA(3,1,2)(0,1,0)[12]	37.28	34.48	44.15	0.16
ARIMA(2,1,0)(0,1,0)[12]	39.55	38.83	43.66	0.19
ARIMA(1,1,0)(0,1,0)[12]	38.36	38.01	41.23	0.19
ARIMA(3,1,0)(1,1,0)[12]	33.58	31.65	39.77	0.16
ARIMA(3,1,0)(0,1,1)[12]	33.54	31.56	39.62	0.16
ARIMA(3,1,0)(1,1,1)[12]	36.35	33.55	43.22	0.16
ARIMA(4,1,0)(0,1,0)[12]	34.73	32.88	40.85	0.16
ARIMA(4,1,1)(0,1,0)[12]	37.6	34.8	44.46	0.16

Appendix 1 Table 23. Diagnostic statistics of candidate ARIMA models of Shigellosis incidences, assessed with out-of-sample validation.

Model	MAE	RMSE	Theil's U
ARIMA(3,1,0)(0,1,0)[12]	0.82	1.04	0.57
ARIMA(3,1,1)(0,1,0)[12]	0.79	0.93	0.51
ARIMA(3,1,2)(0,1,0)[12]	0.83	0.96	0.52
ARIMA(2,1,0)(0,1,0)[12]	0.84	0.96	0.53
ARIMA(1,1,0)(0,1,0)[12]	0.82	1.04	0.54
ARIMA(3,1,0)(1,1,0)[12]	0.81	0.98	0.57
ARIMA(3,1,0)(0,1,1)[12]	0.85	1.08	0.59
ARIMA(3,1,0)(1,1,1)[12]	0.81	0.97	0.53
ARIMA(4,1,0)(0,1,0)[12]	0.83	0.97	0.53
ARIMA(4,1,1)(0,1,0)[12]	0.82	0.97	0.53

Appendix 1 Table 24. Parameters and diagnostic statistics of candidate ARIMA models of Hepatitis A incidences.

Model	AICc	AIC	BIC	RMSE
ARIMA(4,1,0)	−53.46	−54.86	−45.44	18.78
ARIMA(4,1,1)	−51.17	−53.17	−41.82	18.54
ARIMA(4,1,2)	−49.74	−52.48	−39.23	17.74
ARIMA(5,1,0)	−51.77	−53.77	−42.42	17.83
ARIMA(4,2,0)	−45.73	−47.16	−37.82	22.49
ARIMA(3,1,0)	−52.91	−53.82	−46.25	19.75
ARIMA(3,1,1)	−53.13	−54.53	−45.07	18.85
ARIMA(3,1,2)	−52.08	−54.08	−42.73	17.47
ARIMA(4,0,0)	−48.93	−50.88	−39.41	20.56
ARIMA(5,1,1)	−52.33	−55.06	−41.82	15.99

Appendix 1 Table 25. Diagnostic statistics of candidate ARIMA models of Hepatitis A incidences, assessed with out-of-sample validation.

Model	MAE	RMSE	Theil's U
ARIMA(4,1,0)	47.54	45.44	0.85
ARIMA(4,1,1)	49.69	41.82	0.83
ARIMA(4,1,2)	45.22	39.23	0.82
ARIMA(5,1,0)	46.99	42.42	0.84
ARIMA(4,2,0)	84.05	37.82	0.88
ARIMA(3,1,0)	47.54	46.25	0.85
ARIMA(3,1,1)	45.73	45.07	0.83
ARIMA(3,1,2)	50.89	42.73	0.83
ARIMA(4,0,0)	50.92	39.41	0.91
ARIMA(5,1,1)	44.33	41.82	0.88

Appendix 1 Table 26. Parameters and diagnostic statistics of candidate ARIMA models of Enterohemorrhagic *E. coli* incidences.

Model	AICc	AIC	BIC	RMSE
ARIMA(1,0,0)(0,1,0)[12]	2.47	2.13	5.45	0.32
ARIMA(1,0,1)(0,1,0)[12]	4.62	3.92	8.83	0.32
ARIMA(1,1,1)(0,1,0)[12]	6.67	5.95	10.78	0.33
ARIMA(1,0,0)(1,1,0)[12]	3.1	2.39	7.32	0.29
ARIMA(1,0,1)(1,1,0)[12]	2.82	1.61	8.15	0.24
ARIMA(0,0,0)(0,1,0)[12]	9.07	8.96	10.63	0.38
ARIMA(2,0,0)(0,1,0)[12]	4.57	3.87	8.78	0.16
ARIMA(2,0,1)(0,1,0)[12]	6.34	5.13	11.68	0.31
ARIMA(1,0,1)(1,1,1)[12]	9.13	7.13	15.31	0.31
ARIMA(1,0,0)(0,2,0)[12]	28.73	97.28	31.22	0.47

Appendix 1 Table 27. Diagnostic statistics of candidate ARIMA models of Enterohemorrhagic *E. coli* incidences, assessed with out-of-sample validation.

Model	MAE	RMSE	Theil's U
ARIMA(1,0,0)(0,1,0)[12]	2.49	3.64	0.59
ARIMA(1,0,1)(0,1,0)[12]	2.77	4.31	0.71
ARIMA(1,1,1)(0,1,0)[12]	2.51	3.78	0.62
ARIMA(1,0,0)(1,1,0)[12]	2.81	4.33	0.71
ARIMA(1,0,1)(1,1,0)[12]	2.77	4.29	0.71
ARIMA(0,0,0)(0,1,0)[12]	2.79	4.32	0.71
ARIMA(2,0,0)(0,1,0)[12]	2.69	4.21	0.69
ARIMA(2,0,1)(0,1,0)[12]	2.74	4.24	0.73
ARIMA(2,0,2)(0,1,0)[12]	2.73	4.23	0.72
ARIMA(1,0,0)(0,2,0)[12]	2.95	4.46	0.73

Appendix 1 Table 28. Parameters and diagnostic statistics of candidate ARIMA models of Bovine tuberculosis incidences.

Model	AICc	AIC	BIC	RMSE
ARIMA(0,0,2)	−21.6	−24.49	18.84	40.37
ARIMA(1,0,2)(1,0,0)[12]	−21.3	−23.26	−11.78	40.49
ARIMA(1,0,2)(1,0,1)[12]	−20.36	−23.03	−9.64	37.47
ARIMA(1,0,2)(2,0,1)[12]	−21.02	−24.53	−9.24	35.02
ARIMA(1,0,3)(2,0,1)[12]	−18.05	−22.55	−5.34	34.54
ARIMA(1,0,3)(2,0,2)[12]	−15.77	−21.41	−2.29	33.64
ARIMA(2,0,2)(2,0,1)[12]	−18.15	−22.65	−5.44	33.19
ARIMA(2,0,2)(2,0,2)[12]	−15.95	−21.6	−2.48	31.56
ARIMA(2,0,2)(2,0,3)[12]	−11.66	−18.61	2.42	37.07
ARIMA(2,0,1)(2,0,3)[12]	−4.24	−9.88	9.24	36.74

Appendix 1 Table 29. Diagnostic statistics of candidate ARIMA models of Bovine tuberculosis incidences, assessed with out-of-sample validation.

Model	MAE	RMSE	Theil's U
ARIMA(0,0,2)	127.91	139.35	0.64
ARIMA(1,0,2)(1,0,0)[12]	133.39	145.05	0.66
ARIMA(1,0,2)(1,0,1)[12]	136.75	148.02	0.65
ARIMA(1,0,2)(2,0,1)[12]	130.83	142.32	0.64
ARIMA(1,0,3)(2,0,1)[12]	137.29	149.52	0.68
ARIMA(1,0,3)(2,0,2)[12]	138.03	148.79	0.68
ARIMA(2,0,2)(2,0,1)[12]	137.38	149.09	0.67
ARIMA(2,0,2)(2,0,2)[12]	137.64	150.72	0.68
ARIMA(2,0,2)(2,0,3)[12]	139.11	158.75	0.69
ARIMA(2,0,1)(2,0,3)[12]	145.18	158.98	0.72

Appendix 1 Table 30. Parameters and diagnostic statistics of candidate ARIMA models of Bovine brucellosis incidences.

Model	AICc	AIC	BIC	RMSE
ARIMA(0,0,2)	17.08	16.19	23.84	12.46
ARIMA(1,0,2)(1,0,0)[12]	20.10	18.14	29.62	11.97
ARIMA(1,0,2)(2,0,1)[12]	22.44	18.93	34.23	11.06
ARIMA(2,0,2)(2,0,1)[12]	19.72	24.22	36.93	10.89
ARIMA(1,0,3)(2,0,1)[12]	20.11	24.61	37.32	11.23
ARIMA(1,0,3)(2,0,2)[12]	20.7	26.34	39.82	9.34
ARIMA(2,0,2)(2,0,1)[12]	19.72	24.22	36.93	10.89
ARIMA(2,0,2)(2,0,2)[12]	20.93	26.57	40.05	9.23
ARIMA(2,0,2)(2,0,3)[12]	29.71	22.76	43.8	9.01
ARIMA(2,0,1)(2,0,3)[12]	42.16	47.8	61.28	13.14

Appendix 1 Table 31. Diagnostic statistics of candidate ARIMA models of Bovine brucellosis incidences, assessed with out-of-sample validation.

Model	MAE	RMSE	Theil's U
ARIMA(0,0,2)	24.37	25.42	0.61
ARIMA(1,0,2)(1,0,0)[12]	22.62	25.76	0.61
ARIMA(1,0,2)(2,0,1)[12]	21.74	25.28	0.61
ARIMA(1,0,2)(2,0,1)[12]	22.52	26.49	0.63
ARIMA(1,0,3)(2,0,1)[12]	21.99	26.12	0.61
ARIMA(1,0,3)(2,0,2)[12]	22.74	26.34	0.63
ARIMA(2,0,2)(2,0,1)[12]	22.59	24.22	0.63
ARIMA(2,0,2)(2,0,2)[12]	22.66	26.57	0.65
ARIMA(2,0,2)(2,0,3)[12]	23.01	25.76	0.63
ARIMA(2,0,1)(2,0,3)[12]	23.08	27.82	0.66

Appendix 1 Table 32. The number of livestock tested for bovine tuberculosis infection and brucellosis and the budget allocated for the testing in the respective operation*

Value	2017	2018	2019	2020	2021
Number of cattle (1,000 numbers)					
Dairy cow (PPD)	343	342	340	344	340
Beef cattle (Certificate of examination)	462	547	580	625	718
Bovine brucellosis (MRT)	80	77	77	75	75
Bovine brucellosis (RB)	1,938	1,891	1,858	1,824	1,824
Allocated budget (1 USD)					
Dairy cow (PPD)	239,141	238,165	236,771	239,768	253,981
Beef cattle (Certificate of examination)	1,710,510	2,022,790	2,146,000	2,311,020	2,697,240
Bovine brucellosis (MRT)	26,207	25,223	25,092	24,600	24,600
Bovine brucellosis (RB)	654,639	638,931	628,173	616,953	616,953

*PMD, Purified protein derivatives; MRT, Milk ring test; RB. Rose-bengal test

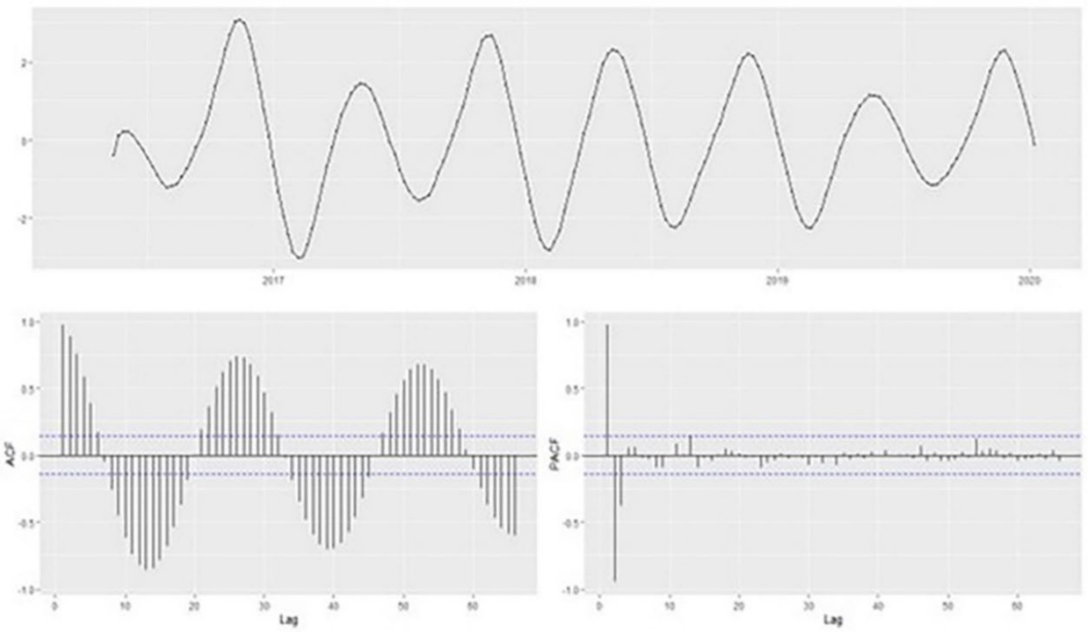

Appendix 1 Figure 1. ACF and PACF plots of Varicella incidences.

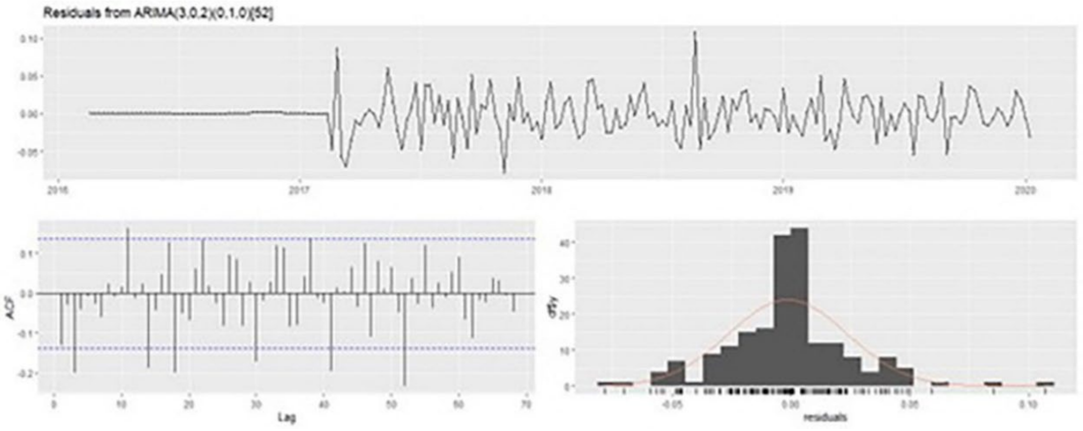

Appendix 1 Figure 2. Residual plots of ARIMA of Varicella incidences.

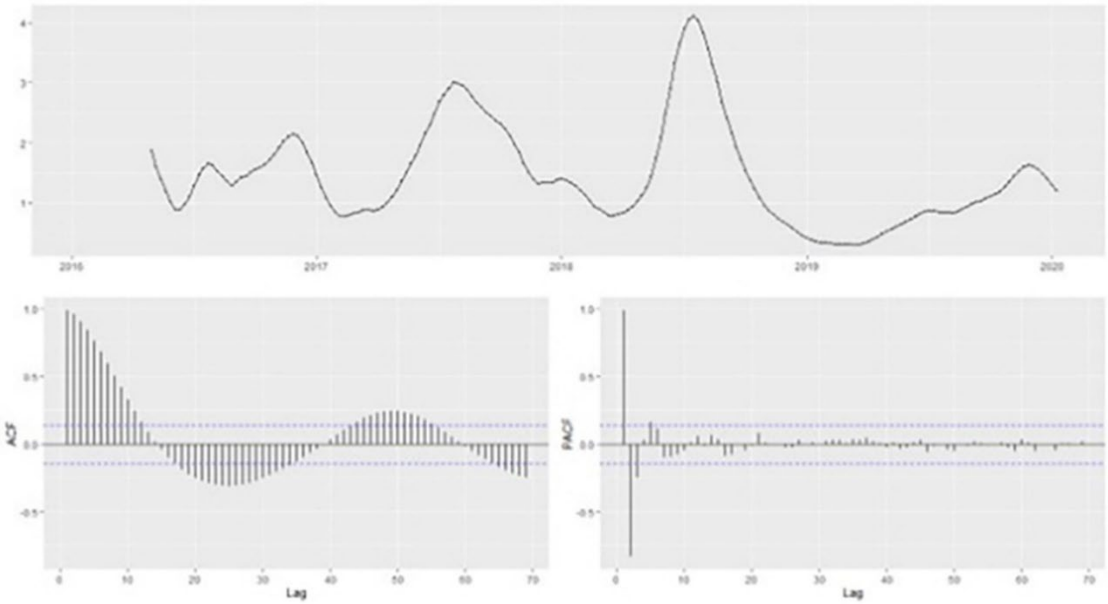

Appendix 1 Figure 3. ACF and PACF plots of Pertussis incidences.

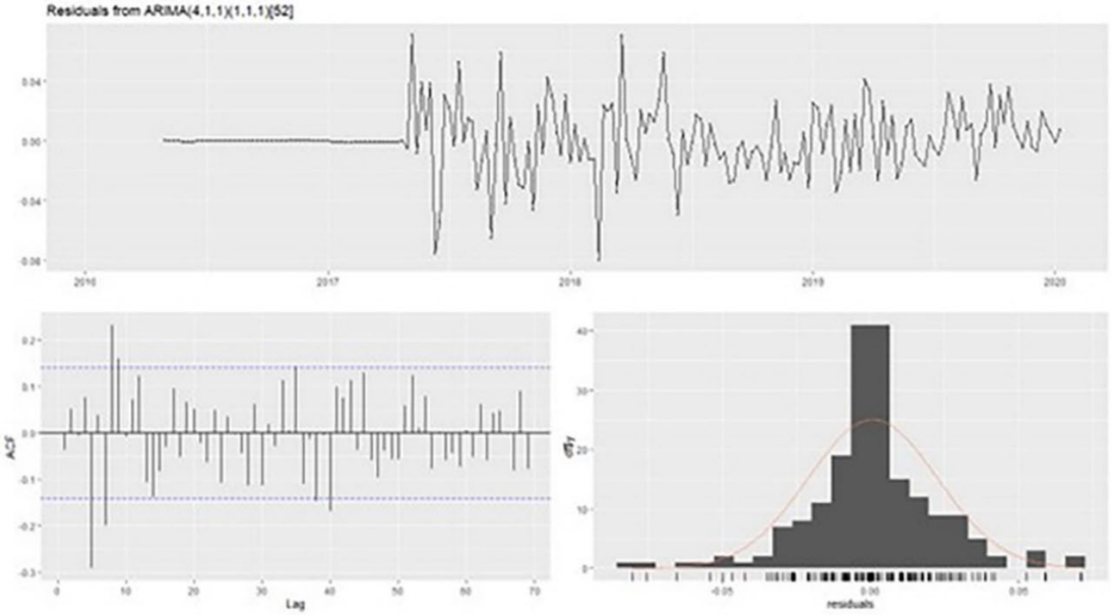

Appendix 1 Figure 4. Residual plots of ARIMA of Pertussis incidences.

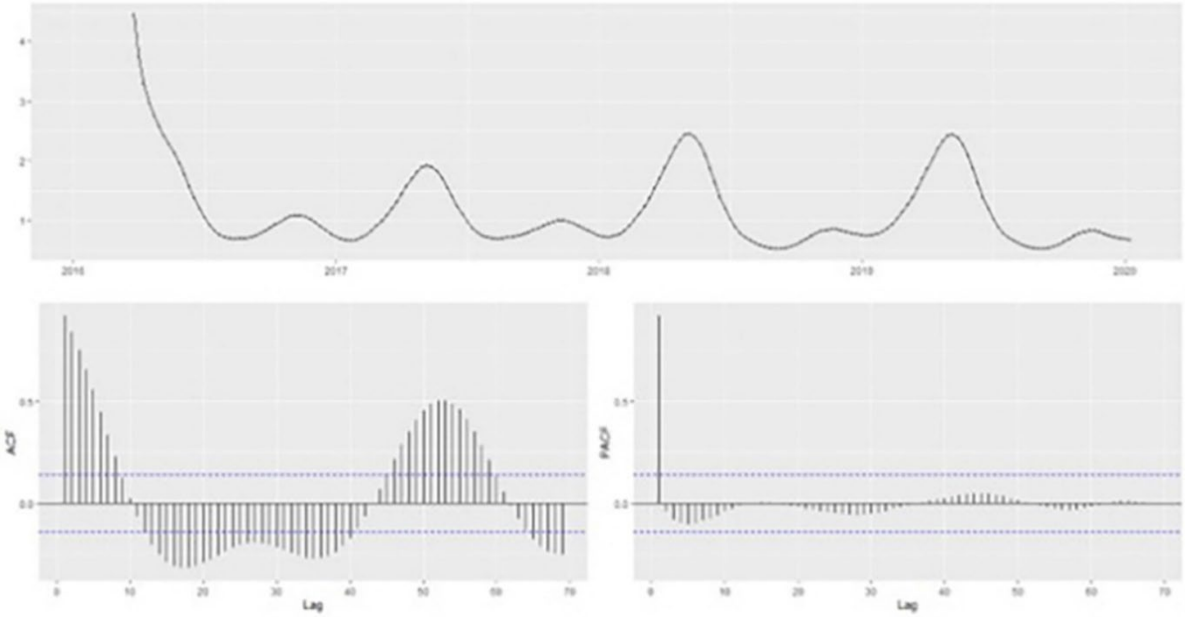

Appendix 1 Figure 5. ACF and PACF plots of Mumps incidences.

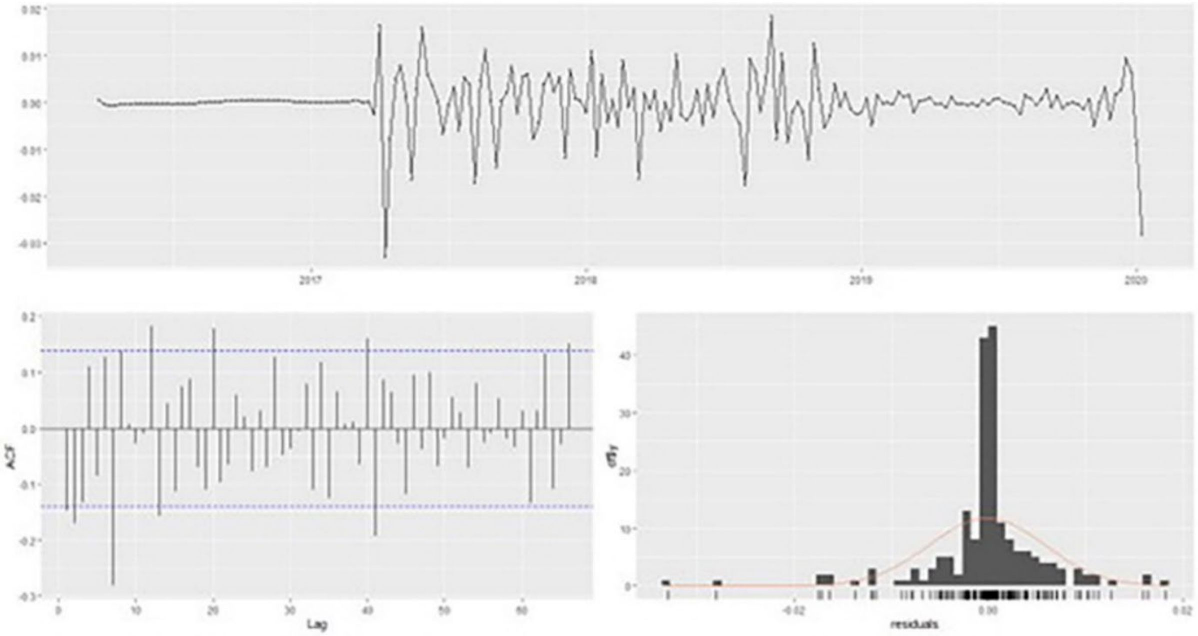

Appendix 1 Figure 6. Residual plots of ARIMA of Mumps incidences.

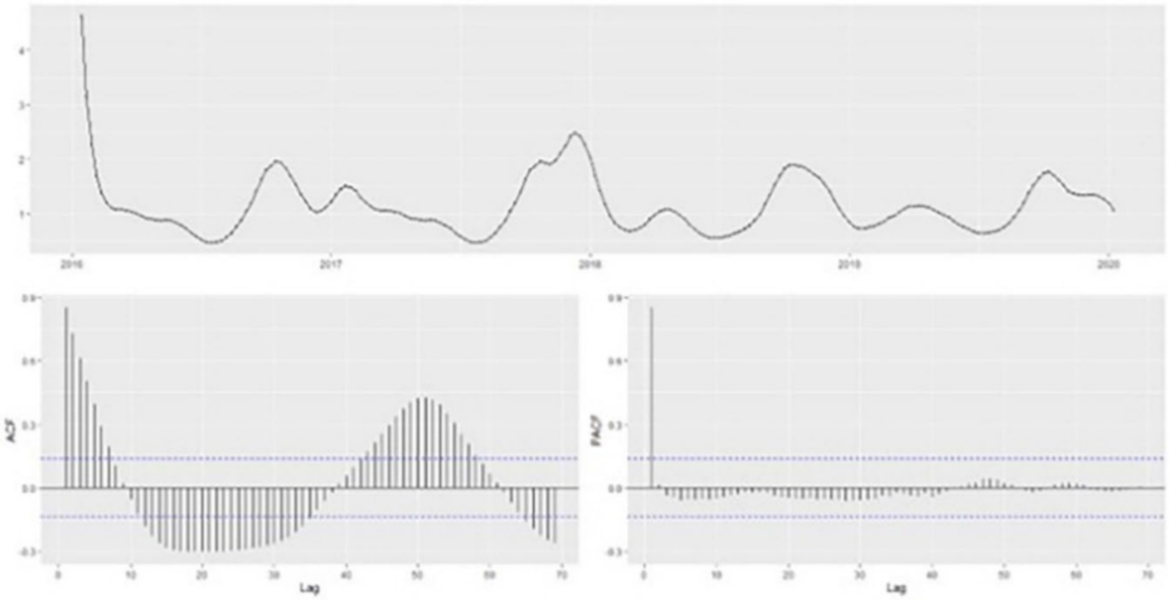

Appendix 1 Figure 7. ACF and PACF plots of invasive pneumococcal disease incidences.

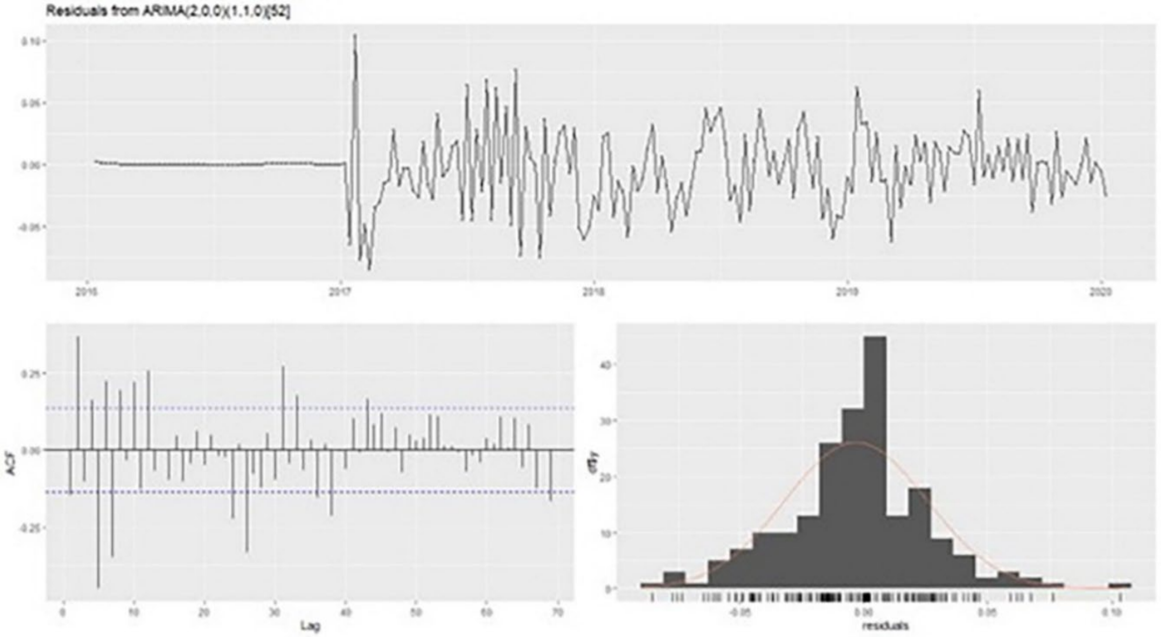

Appendix 1 Figure 8. Residual plots of ARIMA of invasive pneumococcal disease incidences.

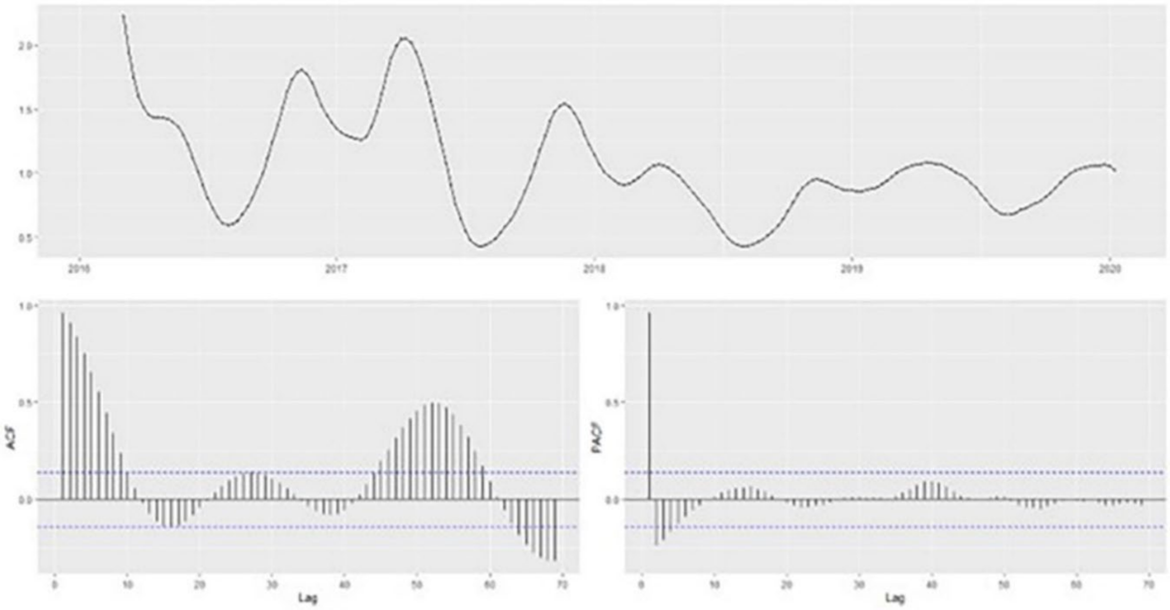

Appendix 1 Figure 9. ACF and PACF plots of scarlet fever incidences.

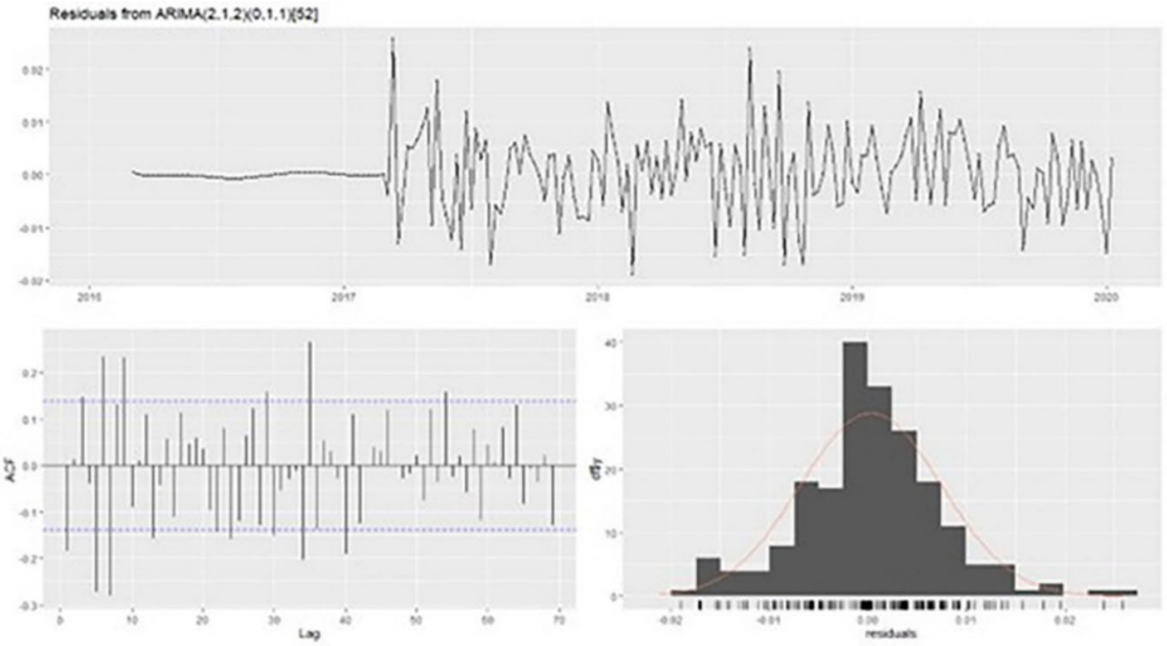

Appendix 1 Figure 10. Residual plots of ARIMA of scarlet fever incidences.

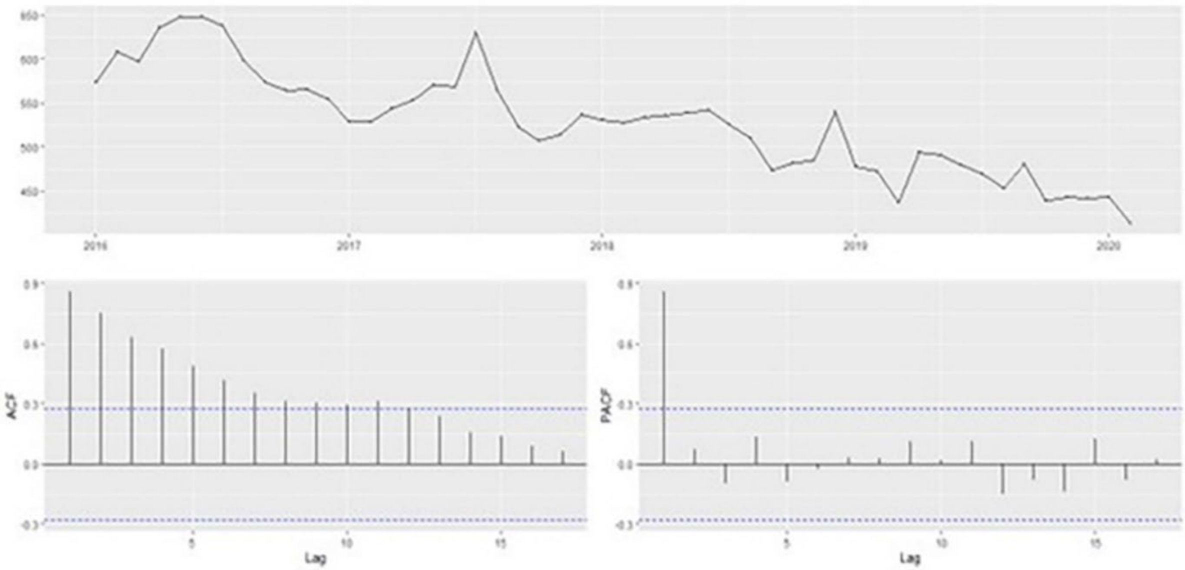

Appendix 1 Figure 11. ACF and PACF plots of Tuberculosis incidences.

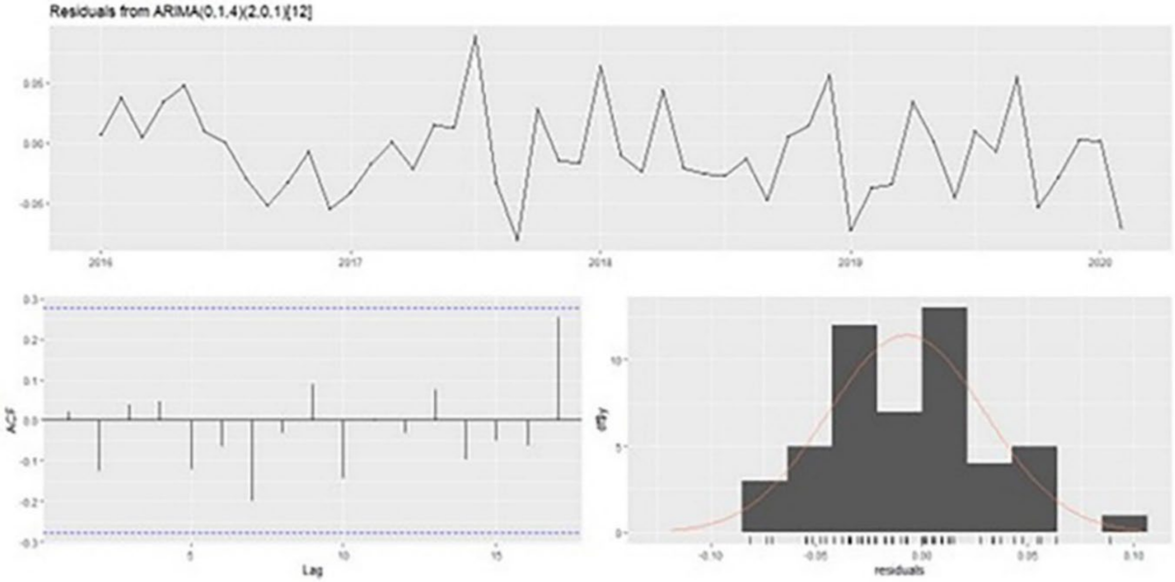

Appendix 1 Figure 12. Residual plots of ARIMA of Tuberculosis incidence

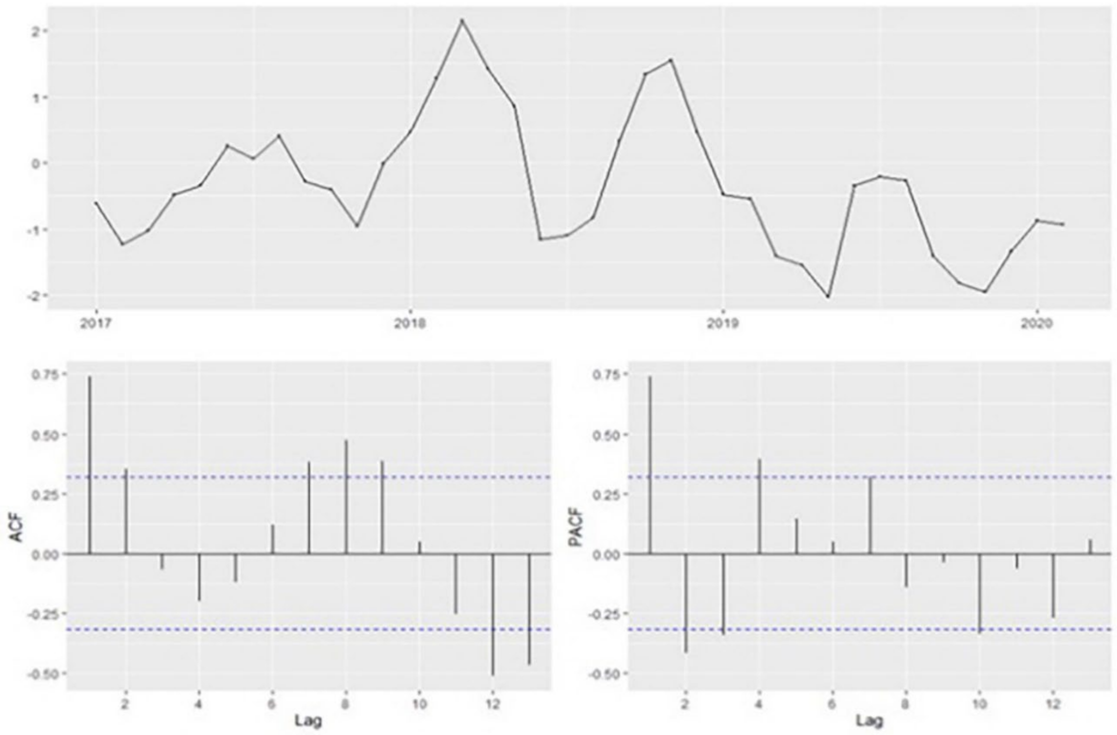

Appendix 1 Figure 13. ACF and PACF plots of Typhoid incidences.

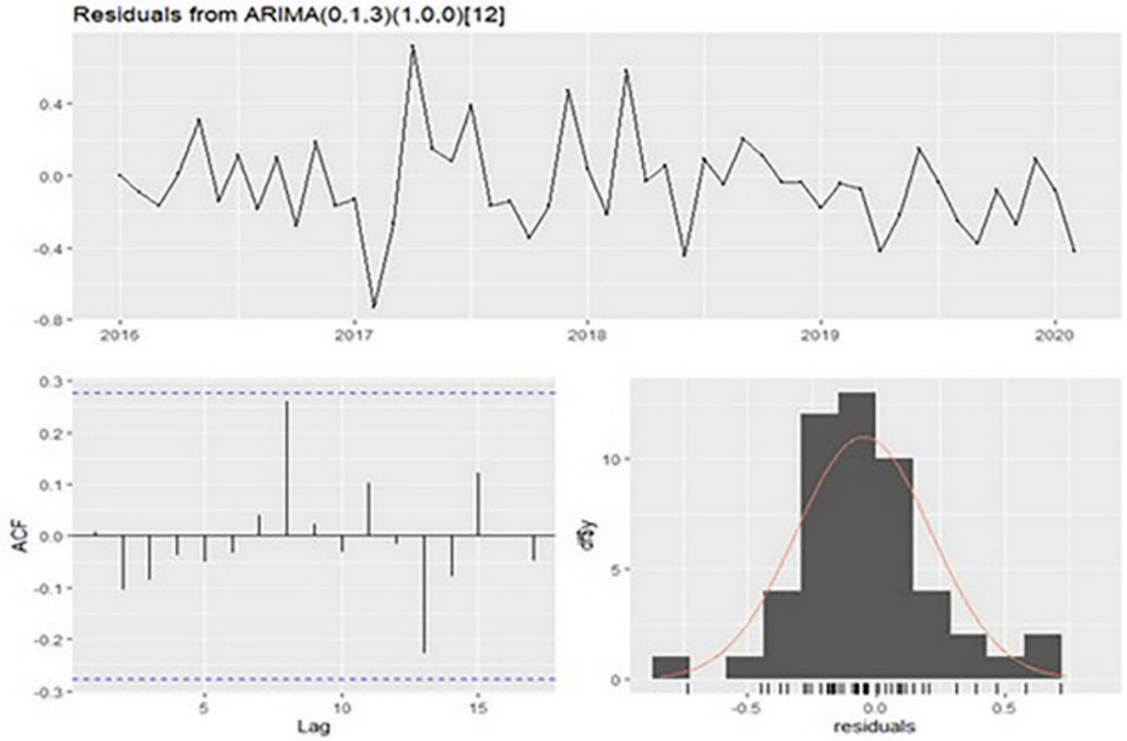

Appendix 1 Figure 14. Residual plots of ARIMA of Typhoid incidences.

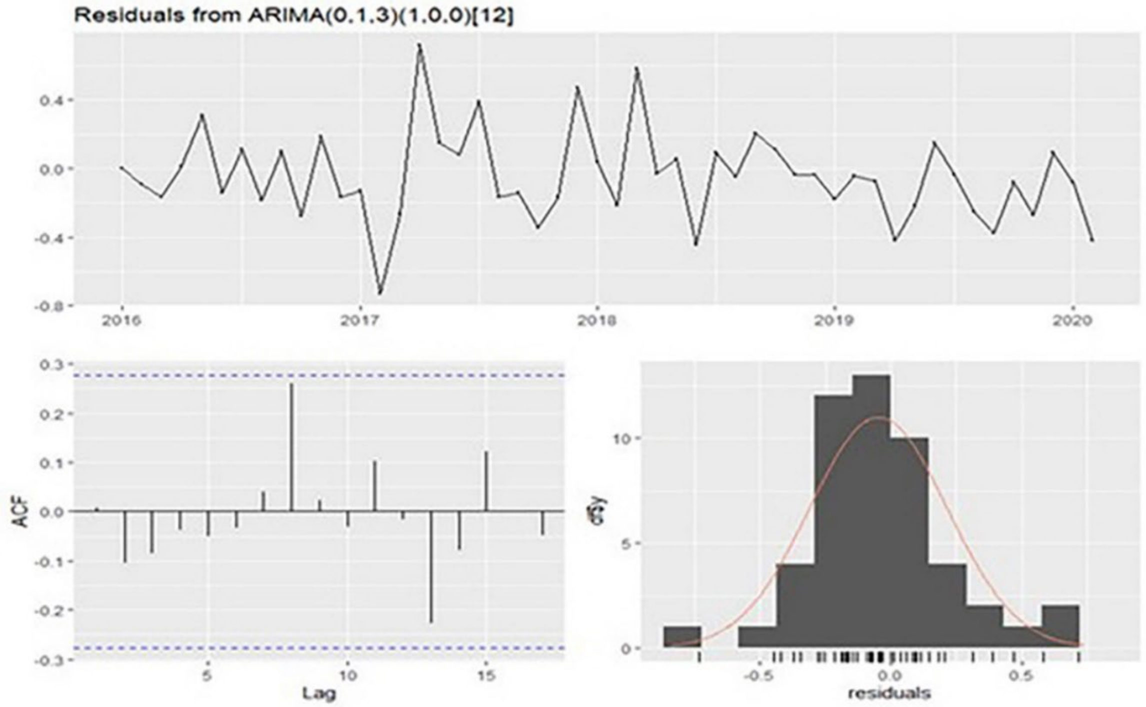

Appendix 1 Figure 15. ACF and PACF plots of Shigellosis incidences.

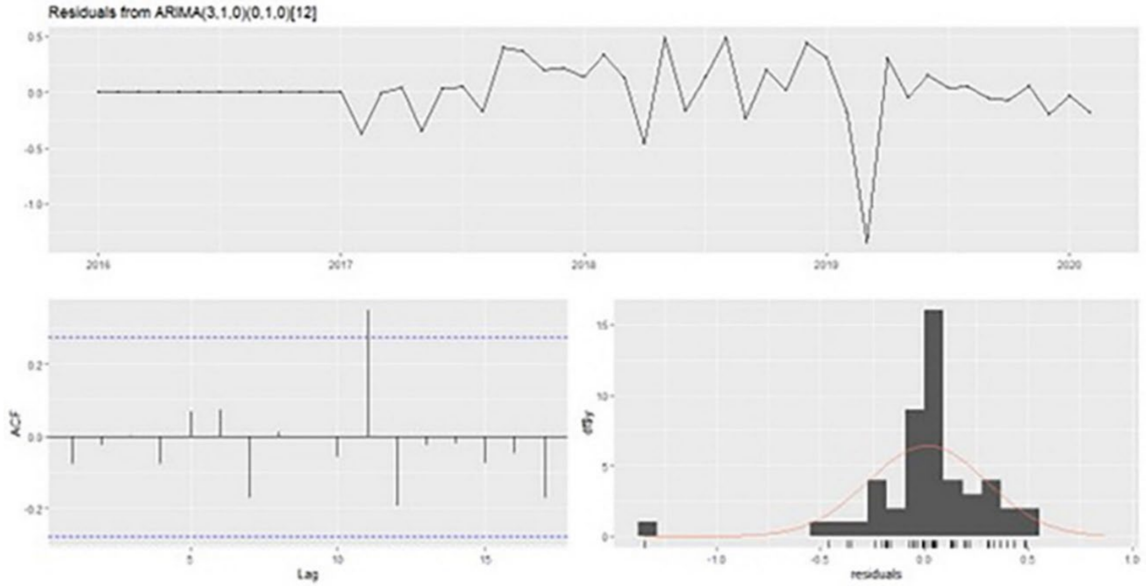

Appendix 1 Figure 16. Residual plots of ARIMA of Shigellosis incidences.

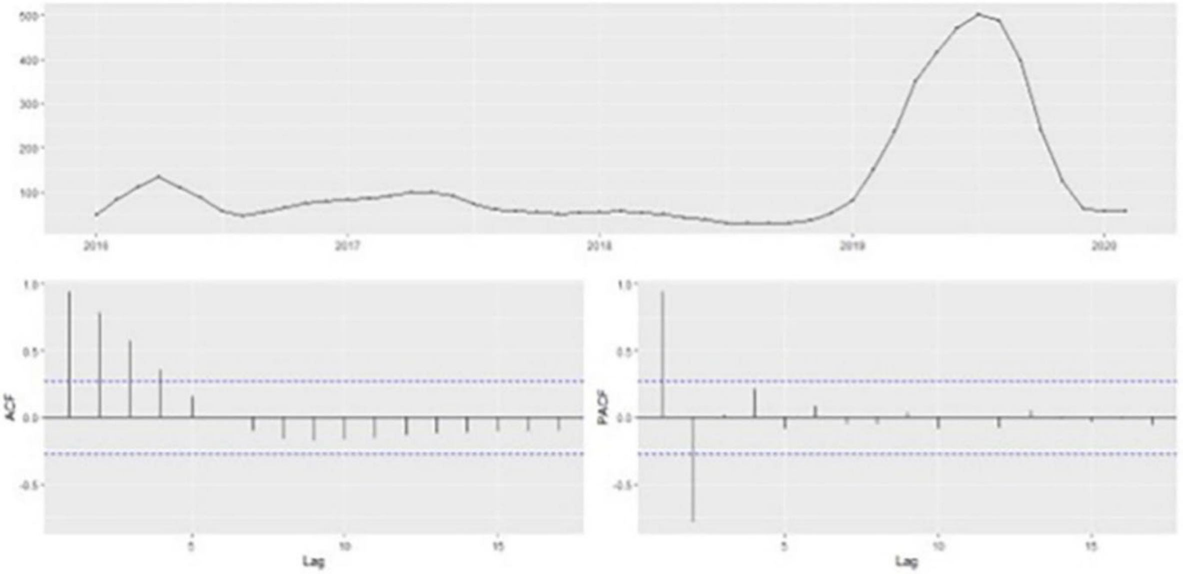

Appendix 1 Figure 17. ACF and PACF plots of Hepatitis A incidences.

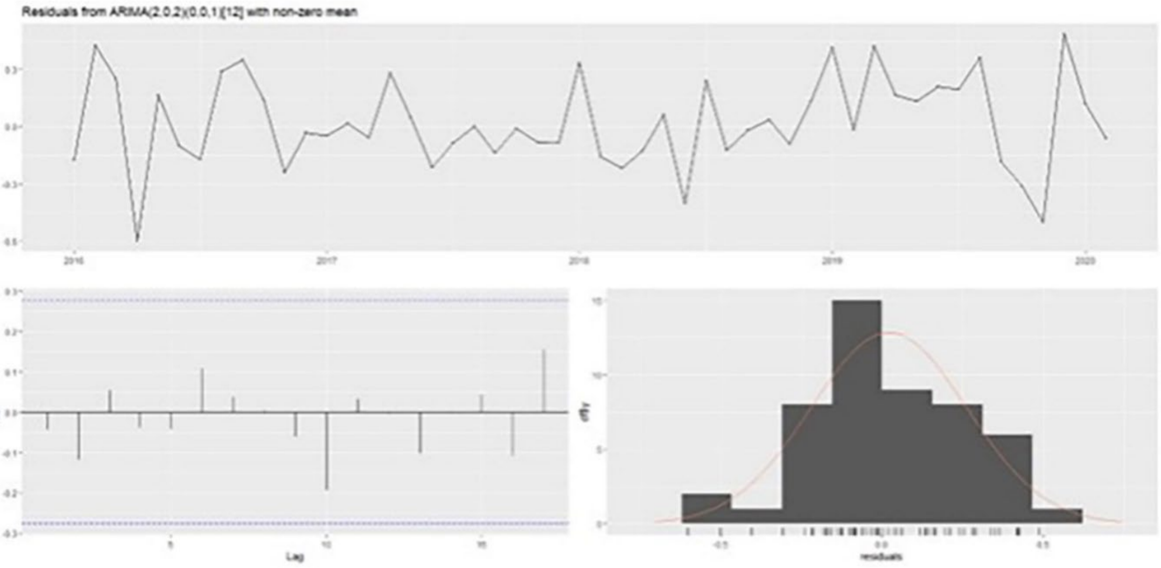

Appendix 1 Figure 18. Residual plots of ARIMA of Hepatitis A incidences.

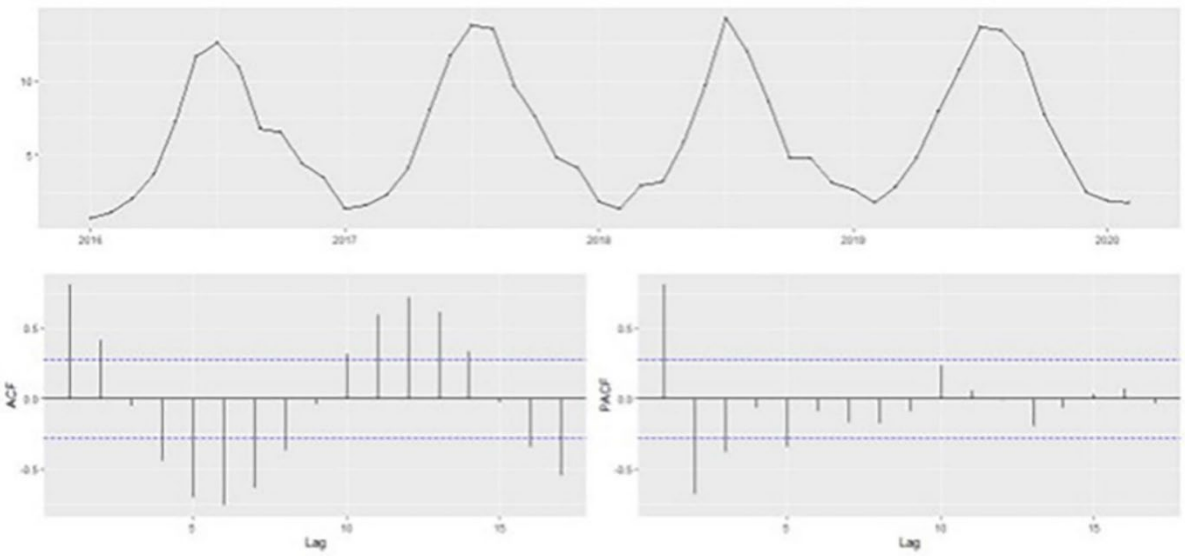

Appendix 1 Figure 19. ACF and PACF plots of Enterohemorrhagic *E. coli* incidences.

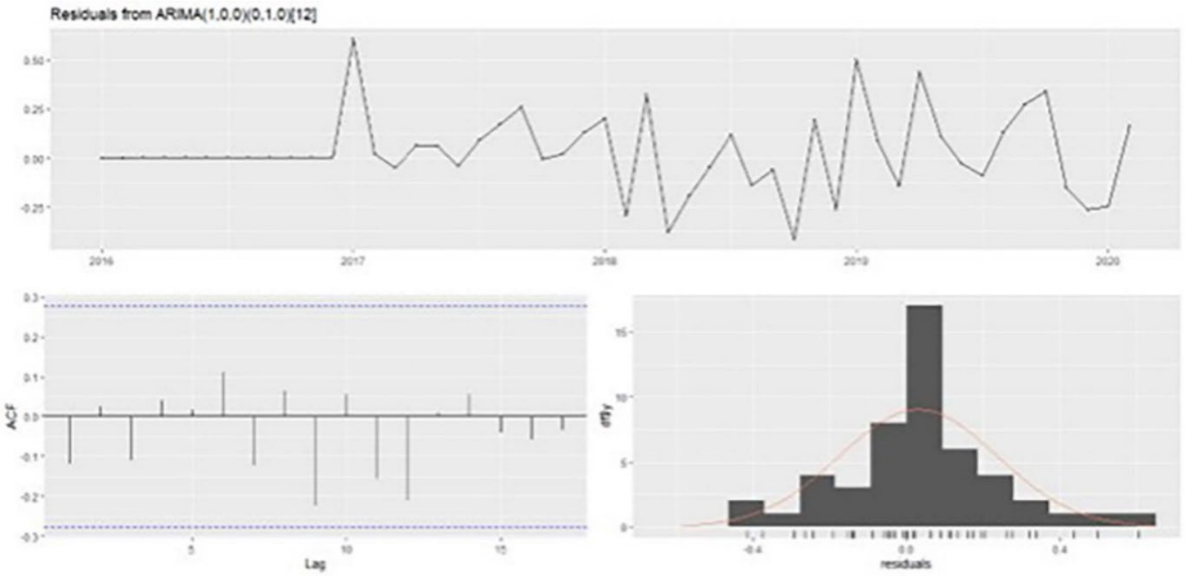

Appendix 1 Figure 20. Residual plots of ARIMA of Enterohemorrhagic *E. coli* incidences.

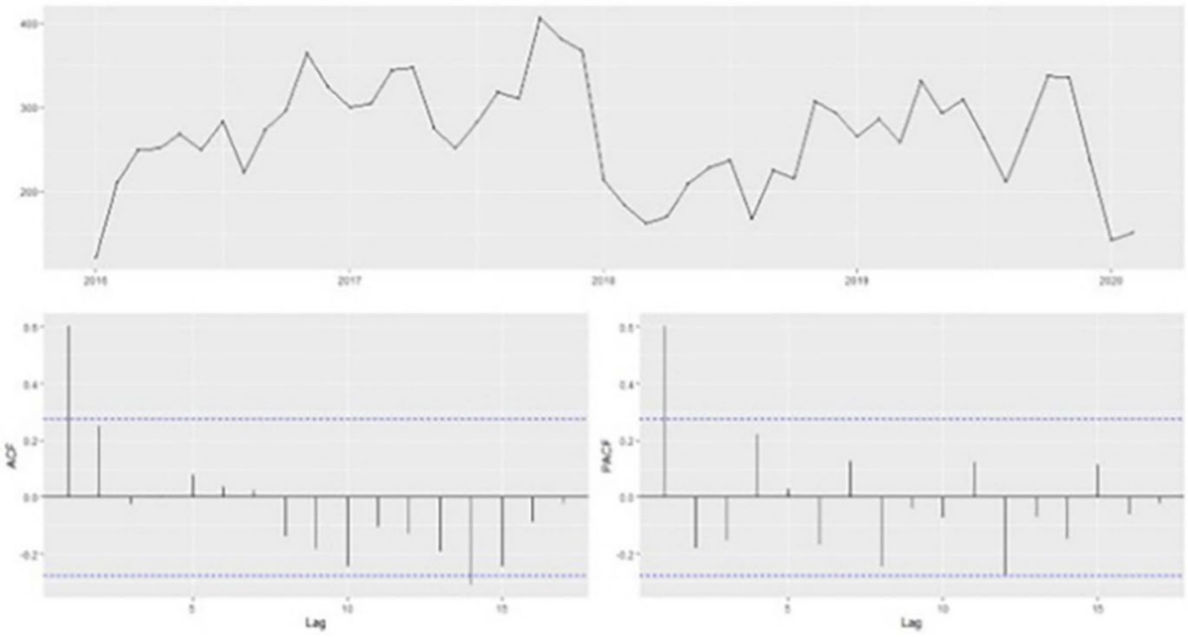

Appendix 1 Figure 21. ACF and PACF plots of Bovine tuberculosis incidences.

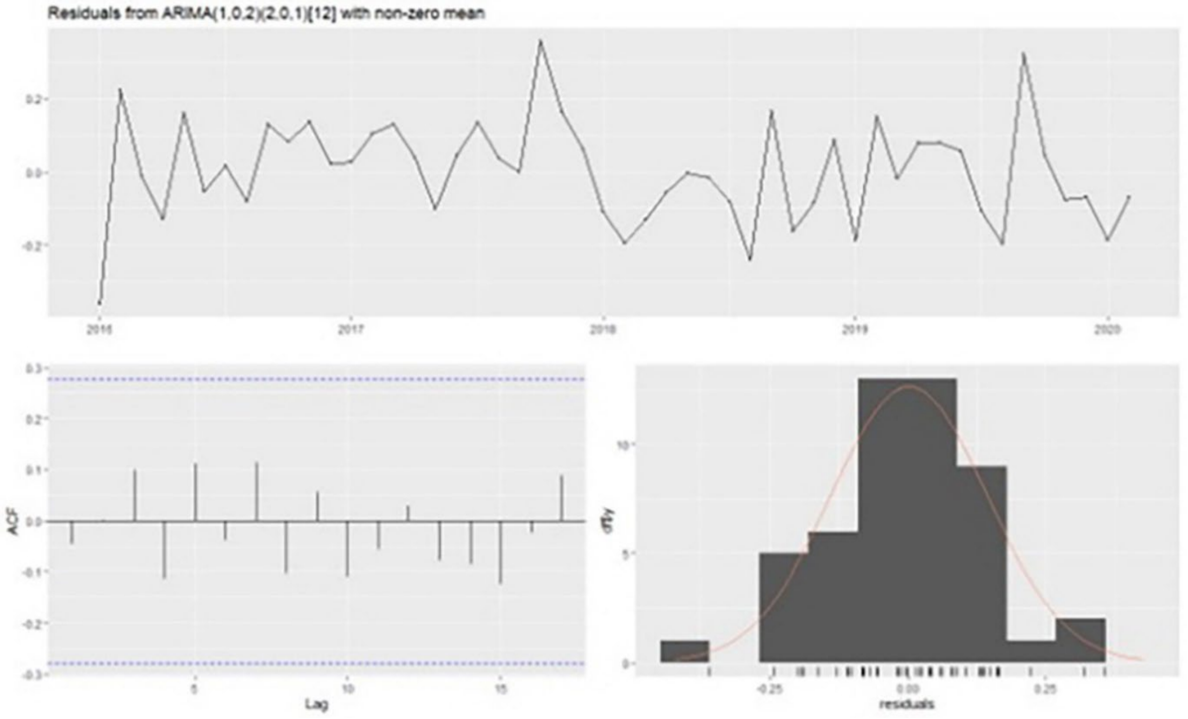

Appendix 1 Figure 22. Residual plots of ARIMA of Bovine tuberculosis incidences.

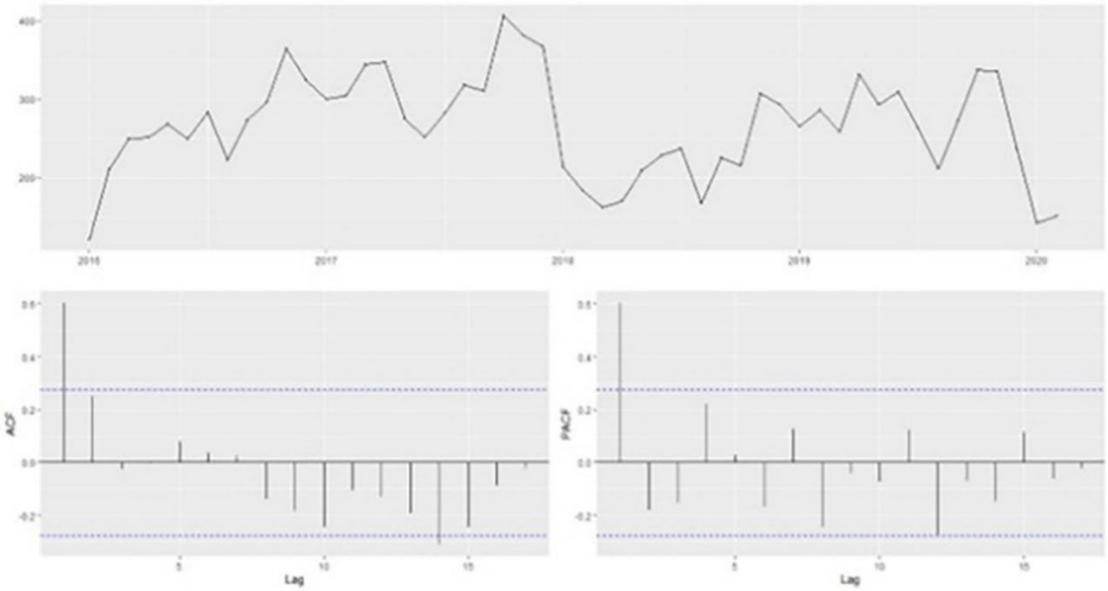

Appendix 1 Figure 23. ACF and PACF plots of Bovine brucellosis incidences.

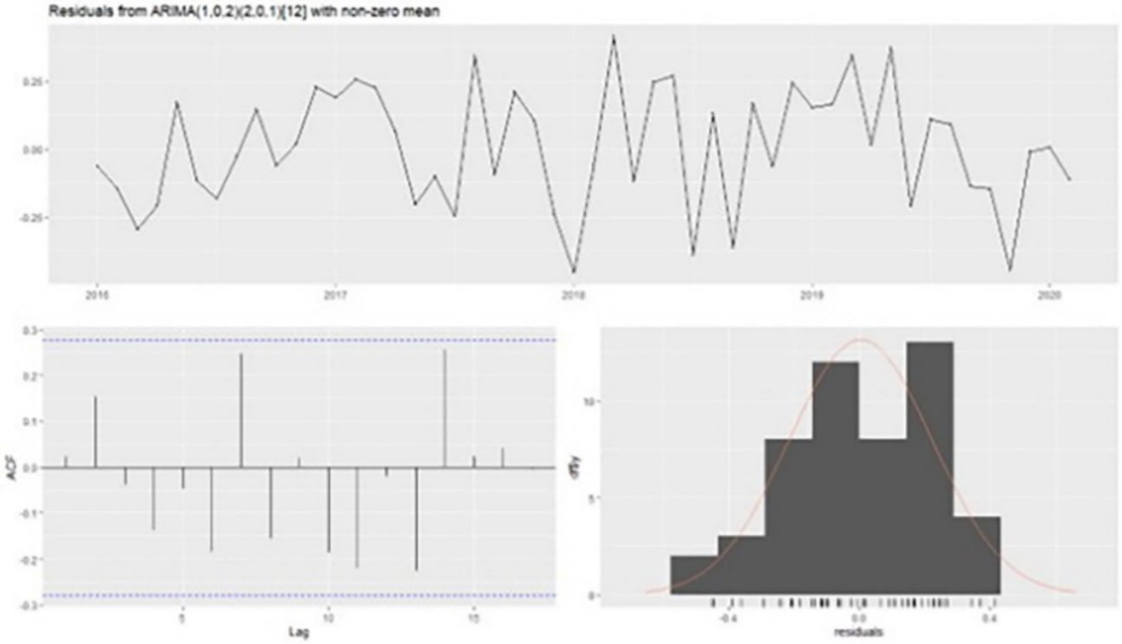

Appendix 1 Figure 24. Residual plots of ARIMA of Bovine brucellosis incidences.
