# Supplementary material for: Trends in Nationally Notifiable Infectious Diseases in Humans and Animals during COVID-19 Pandemic, South Korea
Source: Emerg Infect Dis. 2024 Jun;30(6):1154–63. doi: 10.3201/eid3006.231422 (PMC11138988; doi:10.3201/eid3006.231422)
Supplement: Appendix 2 — Additional data for study of trends in nationally notifiable infectious diseases in humans and animals during the COVID-19 pandemic, South Korea. [file 23-1422-Techapp-s2.pdf]

Article DOI: http://doi.org/10.3201/eid3006.231422

EID cannot ensure accessibility for supplementary materials supplied by authors. Readers who have difficulty accessing supplementary content should contact the authors for assistance.

Trends in Nationally Notifiable Infectious Diseases of Humans and Animals during COVID-19 Pandemic, South Korea

Appendix 2

Reproduction number estimation

The time-dependent reproduction number, R_t, represents the average number of new infections generated by an individual infected with a disease during their infectious period. This time- and context-specific measure is frequently employed to assess the transmissibility of a pathogen during an outbreak. Tracking R_t over time offers insights into how well interventions are working and whether there's a necessity to ramp up control measures (*1*). The primary objective of the control efforts is to lower R_t below the critical threshold of 1, ideally as close to 0 as possible, to effectively manage and control the epidemic. Therefore, in this study, the reproduction number was calculated and used to reflect the dynamics of respiratory infectious diseases more accurately in the process of estimating the effectiveness of NPIs.

The reproduction number, used for analysis of respiratory infectious diseases except tuberculosis, is expressed as shown in a previous study (*2*). The formula is as follows:

$$R_t = \frac{I_t}{\sum_{s=1}^{t} I_{t-s} W(s)}$$

$$W(s) = \left[\frac{1}{\gamma(a)\theta^a}\right] s^{a-1} e^{-\frac{s}{\theta}}$$

where R_t is the reproduction number, t is the number of days elapsed since the start of the epidemic, I_t is the number of cases on day t, $W(s)$ is the current infectivity on day s after infection, a is the shape parameter, and θ is the scale parameter. To estimate current infectivity $W(s)$, we used the serial interval and standard deviation for each disease (Appendix 2 Table) (*3*–

5). After calculating the reproduction number in this manner, it was converted to incidence to estimate the trend of infectious disease occurrence within the population.

The mean serial interval is a crucial epidemiologic variable that describes the infectivity and transmission of an infectious disease. It denotes the average duration between when symptoms first appear in the initial case and when they emerge in a subsequent case (*5*). This interval is extensively employed in the monitoring and management of infectious diseases. The serial interval plays a vital role in epidemic models that assess the effectiveness of intervention strategies and guide policymakers in controlling or mitigating the spread of emerging outbreaks.

Time Series Forecasting with the ARIMA Model

The ARIMA (p, d, q) model is a time series forecasting technique that incorporates elements of autoregressive (AR), moving average (MA), and AR+MA models to make predictions (*6*). ARIMA is commonly used to predict short-term impacts and trends of acute infectious diseases (*6*,*7*). Autoregression in time series data represents how past values influence the current value. The moving average indicates how the prediction errors affect the current value. This component adjusts for the 'noise' or irregularities in the time series by applying past prediction errors to the current value. The parameters p, d, and q in the ARIMA model indicate the order of autoregression, the degree of differencing applied to the original time series, and the order of moving averages, respectively. With respect to some infectious diseases that exhibit seasonality (especially respiratory diseases), we used a seasonal ARIMA (SARIMA [p, d, q] [P, D, Q] s) model. In SARIMA, the additional parameters P, D, Q, and s correspond to seasonal autoregression, seasonal integration, seasonal moving average, and seasonal period length, respectively. Time series forecasting based on the Box-Jenkins method consists of four steps: identification, estimation, diagnostic checking, and forecasting (*8*). We adhered to these steps when making predictions. In addition, we conducted out-of-sample cross-validation to confirm the predictive performance of the model and ensure that the model has not overfitted to the training data. In the validation process, data from the period 2015–2018 was used as training data, and the trend for 2019 was predicted to be compared with observed values.

Appendix 2 Table. Serial intervals of respiratory diseases and their standard deviations.

Respiratory disease	Serial interval (days)	Standard deviation (days)
Varicella	14.0	2.2
Pertussis	22.8	6.5
Mumps	18.0	3.5
Invasive pneumococcal diseases (*Streptococcus pneumoniae*)	6.6	1.8
Scarlet fever	14.0	4.9

References

1. Ferguson NM, Cummings DA, Fraser C, Cajka JC, Cooley PC, Burke DS. Strategies for mitigating an influenza pandemic. Nature. 2006;442:448–52. PubMed https://doi.org/10.1038/nature04795

2. Cori A, Ferguson NM, Fraser C, Cauchemez S. A new framework and software to estimate time-varying reproduction numbers during epidemics. Am J Epidemiol. 2013;178:1505–12. PubMed https://doi.org/10.1093/aje/kwt133

3. Campbell F, Strang C, Ferguson N, Cori A, Jombart T. When are pathogen genome sequences informative of transmission events? PLoS Pathog. 2018;14:e1006885. PubMed https://doi.org/10.1371/journal.ppat.1006885

4. Lau EHY, Nishiura H, Cowling BJ, Ip DKM, Wu JT. Scarlet fever outbreak, Hong Kong, 2011. Emerg Infect Dis. 2012;18:1700–2. PubMed https://doi.org/10.3201/eid1810.120062

5. Vink MA, Bootsma MCJ, Wallinga J. Serial intervals of respiratory infectious diseases: a systematic review and analysis. Am J Epidemiol. 2014;180:865–75. PubMed https://doi.org/10.1093/aje/kwu209

6. Benvenuto D, Giovanetti M, Vassallo L, Angeletti S, Ciccozzi M. Application of the ARIMA model on the COVID-2019 epidemic dataset. Data Brief. 2020;29:105340. PubMed https://doi.org/10.1016/j.dib.2020.105340

7. Geng MJ, Zhang HY, Yu LJ, Lv CL, Wang T, Che TL, et al. Changes in notifiable infectious disease incidence in China during the COVID-19 pandemic. Nat Commun. 2021;12:6923. PubMed https://doi.org/10.1038/s41467-021-27292-7

8. Dritsakis N, Klazoglou P. Forecasting unemployment rates in USA using Box-Jenkins methodology. Int J Econ Financial Issues. 2018;8:9–20.
